# Impact of tissue factor expression and administration routes on thrombosis development induced by mesenchymal stem/stromal cell infusions: re-evaluating the dogma

**DOI:** 10.1186/s13287-023-03582-3

**Published:** 2024-02-27

**Authors:** Van T. Hoang, Duc Son Le, Duc M. Hoang, Trang Thi Kieu Phan, Lan Anh Thi Ngo, Trung Kien Nguyen, Viet Anh Bui, Liem Nguyen Thanh

**Affiliations:** 1 Vinmec Research Institute of Stem Cell and Gene Technology, Vinmec Health Care System, 458 Minh Khai, Hai Ba Trung District, Hanoi, 100000 Vietnam; 2 Center of Applied Science and Regenerative Medicine, Vinmec Health Care System, 458 Minh Khai, Hanoi, 10000 Vietnam; 3 Vinmec International Hospital – Times City, Vinmec Health Care System, 458 Minh Khai, Hanoi, 11622 Vietnam; 4 College of Health Science, VinUniversity, Vinhomes Ocean Park, Gia Lam District, Hanoi, 1310 Vietnam

**Keywords:** Cell therapy, Coagulation, Mesenchymal stem cell, Mesenchymal stromal cell, Tissue factor, Thrombosis, Umbilical cord

## Abstract

**Background:**

Hyperactive coagulation might cause dangerous complications such as portal vein thrombosis and pulmonary embolism after mesenchymal stem/stromal cell (MSC) therapy. Tissue factor (TF), an initiator of the extrinsic coagulation pathway, has been suggested as a predictor of this process.

**Methods:**

The expression of TF and other pro- and anticoagulant genes was analyzed in xeno- and serum-free manufactured MSCs. Furthermore, culture factors affecting its expression in MSCs were investigated. Finally, coagulation tests of fibrinogen, D-dimer, aPPTs, PTs, and TTs were measured in patient serum after umbilical cord (UC)-MSC infusions to challenge a potential connection between TF expression and MSC-induced coagulant activity.

**Results:**

Xeno- and serum-free cultured adipose tissue and UC-derived MSCs expressed the highest level of TF, followed by those from dental pulp, and the lowest expression was observed in MSCs of bone marrow origin. Environmental factors such as cell density, hypoxia, and inflammation impact TF expression, so in vitro analysis might fail to reflect their in vivo behaviors. MSCs also expressed heterogeneous levels of the coagulant factor *COL1A1* and surface phosphatidylserine and anticoagulant factors *TFPI* and *PTGIR*. MSCs of diverse origins induced fibrin clots in healthy plasma that were partially suppressed by an anti-TF inhibitory monoclonal antibody. Furthermore, human umbilical vein endothelial cells exhibited coagulant activity in vitro despite their negative expression of TF and COL1A1. Patients receiving intravenous UC-MSC infusion exhibited a transient increase in D-dimer serum concentration, while this remained stable in the group with intrathecal infusion. There was no correlation between TF expression and D-dimer or other coagulation indicators.

**Conclusions:**

The study suggests that TF cannot be used as a solid biomarker to predict MSC-induced hypercoagulation. Local administration, prophylactic intervention with anticoagulation drugs, and monitoring of coagulation indicators are useful to prevent thrombogenic events in patients receiving MSCs.

*Trial registration* NCT05292625. Registered March 23, 2022, retrospectively registered, https://www.clinicaltrials.gov/ct2/show/NCT05292625?term=NCT05292625&draw=2&rank=1. NCT04919135. Registered June 9, 2021, https://www.clinicaltrials.gov/ct2/show/NCT04919135?term=NCT04919135&draw=2&rank=1.

**Supplementary Information:**

The online version contains supplementary material available at 10.1186/s13287-023-03582-3.

## Background

MSCs are multipotent stem cells found in many tissues, such as adipose tissue (AT), bone marrow (BM), dental pulp (DP), and umbilical cord (UC) [1]. The role of MSCs in regenerative medicine has been widely recognized based on their secretion of growth factors that stimulate new blood vessel formation and tissue/organ regeneration, modulate the immune response, and inhibit inflammatory reactions and their in vivo differentiation into bone, cartilage, and fat [1–4]. The number of clinical trials using MSCs for diverse diseases has increased impressively year after year [5]. Most studies have demonstrated that interventions are safe in both autologous and allogeneic settings [6]. However, some safety issues remain to be further investigated, especially for MSCs originating from tissues other than bone marrow. Several studies have reported that when exposed to blood, MSCs might activate coagulation and increase the risk of thrombosis in patients undergoing stem cell therapy [7–12]. Jung et al. observed pulmonary embolism and infarct in a family after they were treated with autologous AT-MSCs [7]. A 41-year-old patient experienced multiple emboli in the small pulmonary artery branches of both lungs with right pleural effusion after three intravenous infusions of AT-derived stem cells to treat a cervical herniated intervertebral disk. His parents also received a similar therapy for knee osteoarthritis five times. Both of them suffered from multiple emboli in the lungs in concordance with hypercoagulation and mild elevation of D-dimer [7]. Two other patients with chronic renal failure were diagnosed with venous thromboembolism after infusion of intravenous UC-MSCs [8]. They experienced swelling and pain in the forearm after the infusion. Venous blood clots were detected near the puncture site by Doppler ultrasound. In a phase 1b trial using placental-derived MSCs to treat Crohn's disease, one of three patients developed venous thromboses at the site of infusion [10].

The thrombogenic activity of MSCs was described to be associated with tissue factor (TF, also known as tissue marker, CD142, or coagulation factor III), as its expression level can predict vascular inflammation and thrombus formation when cells are transfused into the circulatory system [13–16]. TF plays a central role in the extrinsic coagulation pathway [17, 18]. During the initiation of coagulation, it recruits the factor (F)VII/FVIIa to the plasma membrane and enables an interaction between FVIIa and its substrates FIX and FX [18]. Under normal conditions, inactive TF is expressed abundantly on many cells surrounding blood vessels. Once injury occurs, TF is exposed to its substrates in blood and intermediately initiates an extrinsic coagulation cascade. TF forms a complex with FX and FVII/FVIIa so that FVIIa can cleave FX into active FXa [19]. Additionally, FVIIa can also activate FIX, which forms a complex with FVIIIa to promote FXa accumulation [20]. The protease FXa proteolyzes prothrombin into thrombin. Thrombin also further activates FV, FVIII and platelets during the amplification phase. Active thrombin proteolyzes fibrinogen into soluble fibrin, which generates insoluble fibrin to form fibrin clots to close the injury site [21, 22].

TF has been implicated in inducing thrombosis and venous thromboembolism in response to cell therapy in animal models [13, 15, 23] and humans [24, 25]. They are expressed in cells that are generally not exposed to blood flow, such as subendothelial cells (e.g., smooth muscle cells), perivascular cells, and fibroblasts. MSCs have been shown to heterogeneously express TF [25], depending on tissue sources and culture conditions [26–28]. Serum also has a critical impact on TF expression [27]. Because previous studies were performed mainly on FBS-cultured MSCs, data on serum-free cultured MSCs remain elusive. This study aims to study the TF expression and coagulant activity of xeno- and serum-free cultured MSCs from different tissue sources. Next, the effect of culture and preservation conditions on TF expression was addressed. Finally, the authors analyzed coagulation parameters of patients receiving intravenous and intrathecal xeno- and serum-free cultured UC-MSC infusions and investigated its link with TF expression and administration route.

## Methods

### Primary materials

Human mesenchymal stem/stromal cells (MSCs) derived from AT (*n* = 3), BM (*n* = 3), DP of exfoliated deciduous teeth (*n* = 10), and UC (*n* = 10) samples were isolated and cryopreserved under xeno-free and serum-free conditions as described previously [29]. Peripheral blood mononuclear cells (PBMNCs, *n* = 3) were isolated by gradient centrifugation with Lymphoprep™ (Stem Cell Technologies, Canada). Human umbilical vein endothelial cells (HUVECs, *n* = 3) were extracted from the vein of a UC by enzymatic digestion with collagenase and enriched in an EGMTM-2 Endothelial Cell Growth Medium-2 BulletKitTM (Lonza, USA). All materials were collected after the patients signed an informed consent form. Sample collection and data analysis were approved by the Ethics Committee of Vinmec Healthcare System.

### Cell culture

MSCs from different tissue sources were cultured in StemMACS™ MSC Expansion Media XF (Miltenyi Biotec, Germany) in treated cell culture flasks (NUNC Thermo Scientific, USA) precoated with CellStartTM-coating substrate (Thermo Fisher Scientific, USA) at 37 °C with 5% CO_2_ until the culture reached 80% confluency. The cell morphology was observed under an inverted microscope (Eclipse Ti-S/DS-Fi2-L3, Japan). The cells were harvested using CTS™ TrypLE™ Select Enzyme (Gibco, USA). To examine the population doubling time, MSCs were cultured until passage (P) 10. The population doubling time was calculated at each passage as described previously [29]. For hypoxic culture, MSCs were expanded at 37 °C, 5% CO_2_, and 2% O_2_ in InvivO2 Physiological Cell Culture Workstations (Baker, UK). HUVECs were grown in both EGMTM-2 Endothelial Cell Growth Medium-2 BulletKitTM media (Lonza, USA) at 37 °C with 5% CO_2_ and passaged using CTS™ TrypLE™ Select Enzyme (Gibco, USA).

To analyze the correlation between cell density and TF expression, UC-MSCs were plated at different densities (2000, 8000, and 32,000 cells/cm^2^) on a 6-well plate in duplicate. After two days of culture, the cell density was measured by a Tecan Spark 20 M multimode microplate reader system (Tecan, Switzerland). Cells were then harvested with TrypLE Select Enzyme (Thermo Fisher, USA) and counted, and the same number of cells was stained with CD142-PE antibody (BD Biosciences, USA) for flow cytometry analysis.

### Measurement of clotting time

To examine the clotting time of serum, peripheral blood was collected from healthy donors (*n* = 6) in coagulation sodium citrate tubes. Platelet-poor plasma was collected by centrifugation of anticoagulant blood at 2500 ×*g* for 15 min twice. Plasma samples from six donors were mixed and frozen at − 20 °C for subsequent experiments. MSCs at passage 4 were prepared in NaCl or Ringer’s lactate at a concentration of 50,000 cells/mL. HUVECs and PBMNCs were used as negative controls due to their low coagulant properties in vivo. For each test, 25 μL of platelet-poor plasma was mixed with 50 µL of cell solution in a 2 mL Eppendorf tube and incubated at 37 °C for 5 min. Then, 25 μL of calcium chloride (Shanghai Jizhi Biochemical Technology, China) was added to a final concentration of 5 mM to initiate coagulation. Coagulation time was recorded as the time from the addition of calcium chloride to the formation of a fibrin clot. To minimize technical variations between experiments, a positive control of lysed UC-MSC samples was included in each run and used to normalize the clotting time of the sample of interest.

To block TF activity, UC- and DP-MSCs were incubated with an anti-TF inhibitory monoclonal antibody (clone HTF-1) or an isotype control antibody (clone MOPC-21) (BD Biosciences, USA) at different concentrations (0.05–0.2 µL) for 15 min at 4 °C. The cells were supplemented with 25 μL of platelet-poor plasma and incubated at 37 °C for 5 min. After adding 25 μL of calcium chloride, the coagulation time was measured as described above.

### Treatment of MSCs with cytokines and mitomycin

To stimulate MSCs with inflammatory cytokines, cells were seeded on a 6-well plate at a density of 4000 cells/cm^2^. After two days, the cells were incubated for 6 h in fresh medium supplemented with TNFα and IFNγ (Miltenyi Biotec, Germany) at concentrations of 5 and 10 μg/mL as a single agent or in combination. Cells were then analyzed for TF expression by flow cytometry.

Mitomycin C is a potent DNA crosslinker that inhibits DNA synthesis and cell proliferation. Treatment of cells with mitomycin C led to cell death in a dose-dependent manner. To analyze the effect of cell viability on TF expression, DP-MSCs were incubated with mitomycin C (Sigma, USA) at concentrations of 0, 10, 25, and 50 for 24 h. Cells were then stained with anti-CD142 antibody and 7-AAD for flow cytometry analysis.

### Flow cytometry

For CD142 expression analysis, 10^5^ cells were dissolved in PBS (Gibco, USA) supplemented with 1% FBS (Pan-Biotech, Germany) and stained with CD142-PE antibody (clone HTF-1) or isotype-PE (clone MOPC-21) (BD Biosciences, USA) following the manufacturer’s instructions. The samples were then incubated with 7-AAD (Miltenyi Biotec, Germany) to exclude dead cells. MSCs were costained for MSC-positive markers, including CD73, CD90, and CD105, using a Human MSC Analysis Kit (Becton Dickinson, USA) following the manufacturer’s instructions. The cells were measured by a BD FACSCanto flow cytometer (BD Biosciences) and MACSQuant Analyzer 10 (Miltenyi Biotec, Germany), and data analysis was performed using FlowJo software (BD Biosciences). To assess CD142 expression in the cell population, both TF^+^ cell frequency and median fluorescence intensity (MFI) were scored. The sample MFI was subtracted from the value of the corresponding isotype control to normalize between different measurements.

For the analysis of phosphatidylserine (PS), 10^5^ continuously cultured and cryopreserved/freshly thawed MSCs at passage 3 were suspended in Annexin V binding buffer containing 0.01 M HEPES (Bio Basic, USA), 0.14 M NaCl (STARVISION, VN), and 2.5 mM CaCl_2_ (Shanghai Jizhi Biochemical Technology, China) solution at pH 7.4. Cells were stained with an Annexin V-FITC antibody and 7-AAD (Miltenyi Biotec, Germany) following the manufacturer’s instructions and analyzed using MACSQuant Analyzer 10 (Miltenyi Biotec, Germany).

To examine cell proliferation, 10^6^ cells were fixed with 2X IC fixation buffer (eBioscience™, USA) and permeabilized with 1X permeabilization buffer (eBioscience™, USA) according to the manufacturer’s instructions. Finally, the cells were stained with 7-AAD and analyzed by a BD FACSCanto flow cytometer (BD Biosciences, USA) to evaluate the percentage of cells in the apoptotic SubG1 subset as well as those in the G0/G1 and S/G2/M phases.

### Quantification of active TF

To analyze the activity of TF on MSCs, a colorimetric FXa quantification assay was performed using a human tissue factor activity assay kit (Abcam, UK) following the manufacturer’s instructions. Briefly, 300 cells at passage 3 were suspended in 10 μL and mixed with 50 μL of Assay Diluent, 10 μL of Human FVII, and 10 μL of Human FX. After incubation at 37 °C in a humid incubator for 30 min, 20 μL of FXa substrate was added to reach a final volume of 100 μL. The plate was incubated at 37 °C for 5 min, and the absorbance was measured at 405 nm every 5 min for a total of 30 min using a SpectraMax® M5 Microplate Reader (Molecular Devices LLC, USA). For TF blocking, MSCs were preincubated with 0.1 µg of anti-TF inhibitory antibody (clone HTF-1) or isotype antibody (clone MOPC-21) (BD Biosciences, USA) for 15 min at 4 °C.

### Quantitative real-time PCR

Total RNA was isolated using a QIAamp RNA Blood Mini Kit (Qiagen, Germany) and reverse-transcribed using a SuperScriptTM IV First-Strand Synthesis System (Invitrogen/Thermo Fisher Scientific, USA) following the manufacturer’s instructions. qPCR was performed using PerfeCTa SYBR Green SuperMix (Quantabio, USA) in a 7500 Real-Time PCR System (Applied Biosystems, USA). The forward and reverse primer sequences were as follows: COL1A1 (CCTGGAAAGAATGGAGATGATG and CACCATCCAAACCACTGAAAC); TFF3 (GCTTCAGGCACTACAAATACTG and GCCAAGTACGTCTGCTTCAC); TFPI (CAGTGTGAACGTTTCAAGTATGG and GGGACCGTGAAATTCAAAAAGG); PTGIR (CTGCCATCTTCCTCTGCAAC and TGAAGCAGCGGATCGTGAG); and GAPDH (GGTGTGAACCATGAGAAGTATGA and GAGTCCTTCCACGATACCAAAG). Relative gene expression was normalized to human glyceraldehyde-3-phosphate dehydrogenase (GAPDH). Relative expression was calculated by normalization with a UC-MSC sample using the ΔΔCT method.

### Test of storage conditions

To assess the TF expression of MSCs in infusion solutions for up to 8 h of storage, either fresh or cryopreserved and thawed MSCs were suspended in NaCl solvent (STARVISION, VN) and Ringer lactate (RL, Fresenius Kabi, USA) at a concentration of 2.10^6^ cells/mL. The samples were stored at 4 °C for 0 h, 2 h, 4 h, and 8 h. At each time point, cells were stained with CD142-PE antibody (BD Biosciences, USA) and 7-AAD for flow cytometry analysis and tested for coagulation time.

### Administration of UC-MSCs

Patients diagnosed with stroke and frailty syndromes were enrolled from 2021 to 2022 in phase I/II trials (ClinicalTrials.gov Identifier: NCT05292625 and NCT04919135, respectively). Patients with stroke were randomized into two groups: the first group received UC-MSCs via the intravenous route (*n* = 16), and the other group received UC-MSCs via the intrathecal route (*n* = 16). Those with frailty syndromes were intravenously infused with UC-MSCs (*n* = 19). All patients received two doses of 1.5 million cells/kg body weight, which were administered three months apart. The patients were injected with 40 mg Solumedrol and prophylactically treated with Lovenox 4000 Ui or Xarelto 10 mg 30 and 60 min before cell administration, respectively. UC-MSCs were infused intravenously within 30 min using an automatic electric injection pump. For intrathecal administration, cells were infused through the space between the fourth and fifth lumbar vertebrae using an 18-gauge needle within 30 min under general anesthesia as described previously [30].

### Laboratory tests of coagulation factors

The coagulation activity of patients after UC-MSC infusions was monitored by coagulation tests. For this purpose, blood from patients before, as well as 24 and 48 h after UC-MSC infusions, was collected in coagulation sodium citrate tubes. Fibrinogen and D-dimer were quantified at the ISO 15189 and CAP-certificated laboratory of Vinmec Times City Hospital, Vinmec Healthcare System, using an automated system ACL TOP 500 CTS (Instrumentation Laboratory, US) and STAR Max® (Stago, USA). The activated partial thromboplastin time (aPTT), prothrombin time (PT), and thrombin time (TT) were measured using the same systems.

To evaluate the PT in the presence of MSCs, 10^6^ UC-MSCs at passage 5 were mixed with 2 mL of the patient’s whole blood in a coagulation sodium citrate tube. The mixture of blood and cells was then tested for PT using the ACL TOP 500 CTS system (Instrumentation Laboratory, US).

### Data analysis

Statistical analyses were performed with GraphPad Prism version 8 (https://www.graphpad.com/scientific-software/prism/). ANOVA and Tukey HSD tests were used to evaluate differences between groups. A *p* value < 0.05 was considered significant. Statistical significance is presented as * for 0.01 < *p* < 0.05; ** for 0.001 < *p* < 0.01; *** for 0.0001 < *p* < 0.001; and **** for *p* < 0.0001.

## Results

### Clotting time of healthy plasma in the presence of MSCs

**Fig. 1:**
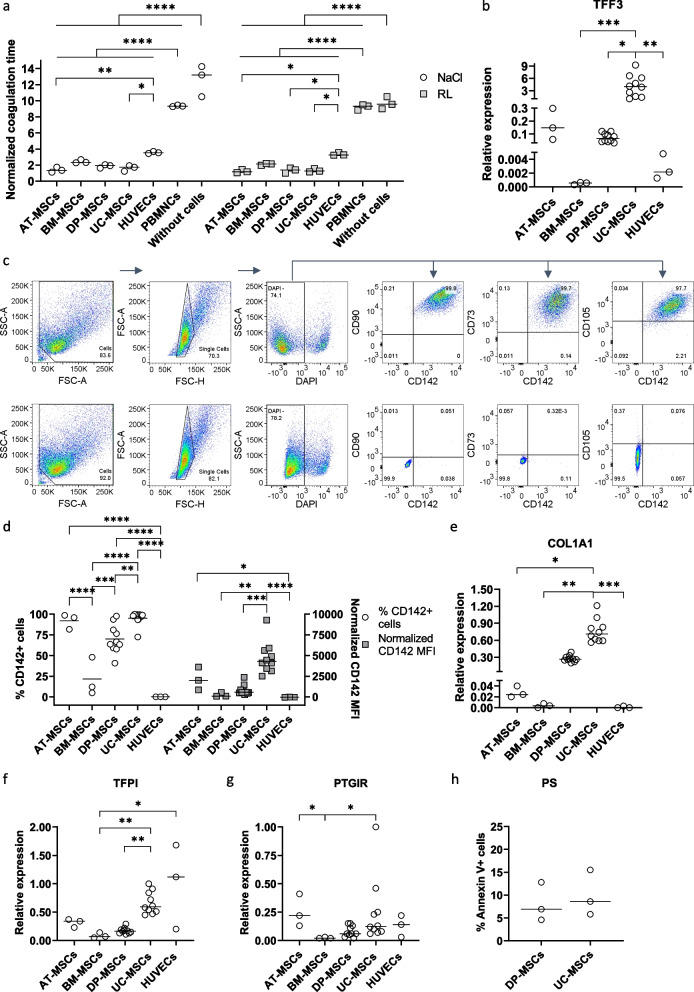
Coagulant activity of xeno- and serum-cultured MSCs and their expression of pro- and anticoagulant factors. **a** MSCs derived from AT, BM, DP, and UC as well as HUVECs and PBMNCs (*n* = 3 each) were prepared in NaCl and RL and supplemented with plasma from healthy donors (*n* = 6) and CaCl_2_ to measure the time required to form fibrin clots. MSCs exhibited the highest coagulant activity, followed by HUVECs, PBMNCs and the negative control without cells. **b** Gene expression analysis of TF revealed the highest expression in UC-MSCs, moderate levels in AT- and DP-MSCs, and low expression in BM-MSCs and HUVECs (UC- and DP-MSCs: *n* = 10 and other cell types: *n* = 3). **c**, **d** TF protein expression was analyzed by flow cytometry. A representative sample of UC-MSCs demonstrated coexpression of TF/CD142 and MSC-positive markers, including CD90, CD73, and CD105 (c). The frequency of TF^+^ cells and CD142 MFI were analyzed, showing a high level of this factor in UC- and AT-MSCs, lower levels in DP- and BM-MSCs, and negative expression in HUVECs (**d**). **e**–**g** Gene expression was analyzed for the procoagulant factor COL1A1 and the anticoagulant factors TFPI and PTGIR. UC-MSCs expressed significantly higher COL1A1 than AT- and BM-MSCs and HUVECs (**e**). In terms of the anticoagulant factors TFPI (**f**) and PTGIR (**g**), UC- and AT-MSCs and HUVECs displayed higher expression than DP- and BM-MSCs. **h** The exposure of the negatively charged phosphatidylserine (PS) in the outer membrane of UC- and DP-MSCs was analyzed using an anti-Annexin V antibody. A subset of UC- and DP-MSCs in culture (continuously cultured cells) were positive for Annexin V

MSCs derived from different origins, including AT, BM, DP, UC, HUVECs, and PBMNCs, were tested for the time required to form clots in healthy plasma in the presence of Ca^2+^ (Fig. 1a). The cells were suspended in either NaCl or Ringer’s lactate, which are two common infusion solutions. The normalized clotting times of AT-, BM-, DP-, and UC-MSCs in NaCl (mean ± SD) were 1.39 ± 0.32, 2.42 ± 0.22, 1.86 ± 0.22, and 1.62 ± 0.35 s, respectively, compared to 3.55 ± 0.13 s for HUVECs (AT- and UC-MSCs versus HUVECs: *p* < 0.01 and *p* < 0.05, respectively), 9.35 ± 0.10 s for PBMNCs (*p* < 0.0001), and 12.65 ± 1.93 s for NaCl (*p* < 0.0001). Similarly, MSC samples from all analyzed sources suspended in Ringer’s lactate indicated a decreased clotting time compared to the negative controls, including HUVECs (AT-MSCs, DP-MSCs and UC-MSCs versus HUVECs: *p* < 0.05), PBMNCs (*p* < 0.0001), and Ringer’s lactate samples (*p* < 0.0001) (Fig. 1a). Among MSCs from different tissue sources, BM-MSCs showed the lowest coagulant activity, followed by DP-MSCs, UC-MSCs, and AT-MSCs (Additional file 2: Fig. S1a).

### TF expression of MSCs derived from diverse sources

The expression of TF was analyzed by real-time PCR in AT-, BM-, DP-, UC-MSCs, and HUVECs. TFF3, which encodes TF, was expressed at different levels depending on MSC tissue origins (Fig. 1b). UC-MSCs showed the highest level of TFF3, followed by AT-MSCs and DP-MSCs, while this factor was hardly expressed in BM-MSCs and HUVECs (UC-MSCs versus BM-MSCs, DP-MSCs, and HUVECs: *p* < 0.001, *p* < 0.05, and *p* < 0.01, respectively).

Next, the surface expression of TF was analyzed by flow cytometry. Figure 1c depicts a representative UC-MSC sample showing coexpression of TF (also named CD142) and MSC-positive markers, including CD73, CD90, and CD105. The expression of TF/CD142 was plotted by the frequency of positive cells and the median fluorescence intensity (MFI) normalized to the isotype controls (Fig. 1d). MSCs expressed diverse levels of this factor, with frequencies of TF-expressing cells (mean ± SD) of 92.17 ± 9.08, 21.66 ± 23.10, 70.05 ± 17.33, and 95.13 ± 8.33% in AT-, BM-, DP-, and UC-MSCs, respectively, compared to 0.03 ± 0.007% in HUVECs, while the MFIs (mean ± SD) were 2148 ± 1390, 232.3 ± 270.3, 806.3 ± 633.3, 4739 ± 1912, and -38.77 ± 8.05 in AT-, BM-, DP-, UC-MSCs, and HUVECs, respectively. UC-MSCs expressed the highest level of TF (TF^+^ frequency: AT- and DP-MSCs vs. BM-MSCs: *p* < 0.001, UC-MSCs vs. BM-MSCs: *p* < 0.0001, DP- MSCs vs. UC-MSCs: *p* < 0.01; TF MFI: BM- vs. UC-MSCs: *p* < 0.01, DP- vs. UC-MSCs: *p* < 0.001).

### Gene expression of other pro- and anticoagulation factors in MSCs

Gene expression of another pro-coagulation factor, collagen 1A1 (COL1A1), and anticoagulation factors, tissue factor pathway inhibitor (TFPI) and prostaglandin I2 receptor (PTGIR), was quantified by real-time PCR (Fig. 1e–g). UC-MSCs increasingly expressed COL1A1 in concordance with TFF3 compared to HUVECs (*p* < 0.001). Among MSCs from different sources, AT- and BM-MSCs exhibited lower expression of COL1A1 than UC-MSCs (*p* < 0.05 and *p* < 0.01, respectively) (Fig. 1e). In terms of anticoagulation factors, TFP1 showed higher expression in HUVECs and UC-MSCs than in BM- and DP-MSCs (BM-MSCs versus HUVECs: *p* < 0.05 and BM- and DP-MSCs versus UC-MSCs: *p* < 0.001) (Fig. 1f). PTGIR expression was higher in AT- and UC-MSCs than in BM-MSCs (both *p* < 0.05) and comparable in other analyzed cell populations (Fig. 1g).

In addition, the expression of PS on the UC- and DP-MSC surfaces was evaluated through its binding ability to Annexin V (Fig. 1h). UC- and DP-MSCs in culture (continuously cultured cells) displayed 8.10 ± 4.23% and 9.97 ± 4.99% positivity for Annexin V, respectively. Cryopreserved and freshly thawed cells showed 11.13 ± 5.88% and 12.20 ± 6.71% Annexin V^+^ cells, respectively (Additional file 2: Figure S1b). Annexin V^+^ 7AAD^−^ cells, which represented an early apoptotic cell subset, expressed diverse CD142 levels from 7.13 ± 6.24% in continuously cultured DP-MSCs to 57.00 ± 11.81% in continuously cultured UC-MSCs (Additional file 2: Fig. S1c, d). Late apoptotic Annexin V^+^ 7AAD^+^ cells were negative for CD142 (Additional file 2: Fig. S1c, d).

### The impact of TF inhibition on FX activation and MSC-mediated coagulation

**Fig. 2:**
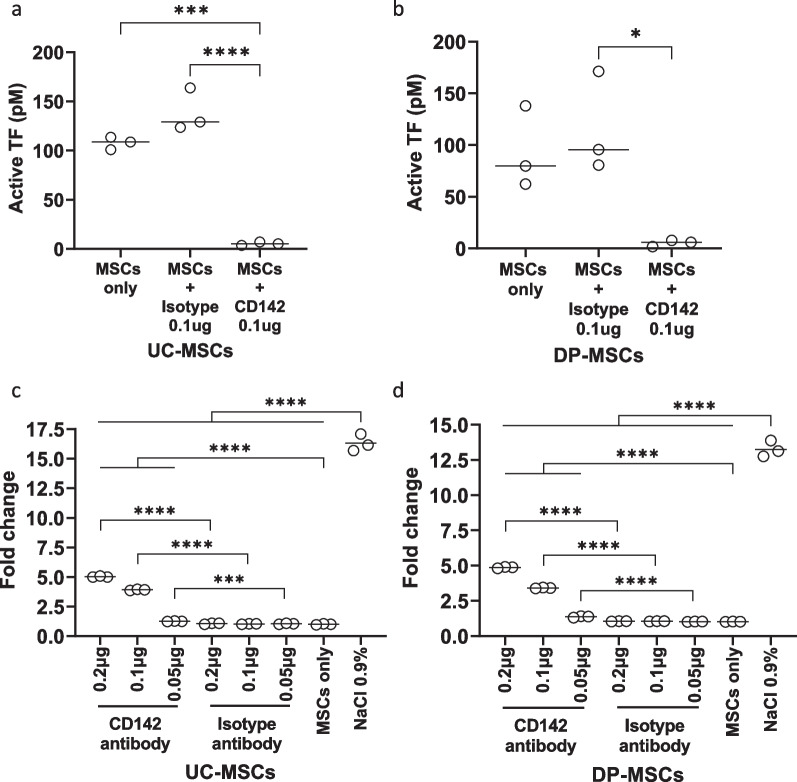
Impact of TF inhibition on the coagulant activity of UC- and DP-MSCs. **a** and **b** UC-MSCs (**a**) and DP-MSCs (**b**) were incubated with an anti-TF inhibitory monoclonal antibody (clone HTF-1) or an isotype control antibody (clone MOPC-21) and analyzed using a colorimetric FXa quantification assay. The HTF-1 antibody was capable of suppressing active TF. **c **and** d** The coagulant activity of UC-MSCs (**c**) and DP-MSCs (**d**) was significantly reduced in the presence of the HTF-1 antibody compared to the isotype control, but it was higher than that of the NaCl negative control samples

UC- and DP-MSCs were treated with either the anti-TF inhibitory monoclonal antibody (clone HTF-1) or the isotype control antibody (clone MOPC-21), and their TF activity was analyzed using a colorimetric FXa quantification assay (Fig. 2a and b, respectively). Active TF levels were significantly decreased in the presence of the anti-TF inhibitory antibody compared to the isotype controls (UC-MSCs: *p* < 0.001 and DP-MSCs: *p* < 0.05). Samples containing only MSCs showed TF activity similar to that of the isotype control.

To test whether MSC-induced coagulation depends on TF, the clotting time of UC- and DP-MSCs was examined in the presence of the anti-TF inhibitory antibody (Fig. 2c and d, respectively). MSC-induced clotting was reduced in the presence of the anti-TF antibody compared to the isotype control samples and those with MSCs only (*p* < 0.0001 for both UC-MSCs and DP-MSCs). However, the clotting time of anti-TF antibody-treated samples was significantly shorter than that of the NaCl negative control samples, which did not contain MSCs (p < 0.0001 for UC-MSCs and DP-MSCs). Similarly, the coagulant activity of AT-MSCs was partly inhibited in the presence of the inhibitory anti-TF antibody (*p* < 0.0001) (Additional file 3: Fig. S2a).

### Analysis of TF expression in UC-MSCs over passages

**Fig. 3:**
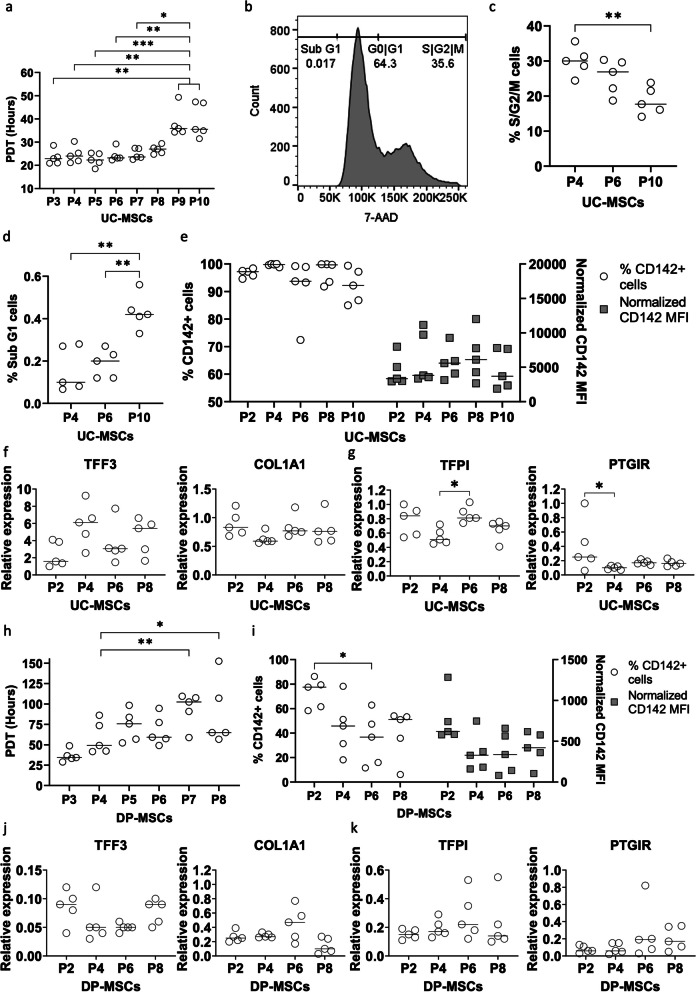
Impact of culture passage on TF expression in UC- and DP-MSCs. **a** The growth ability of UC-MSCs was analyzed over passages from P3 to P10. Their PDT increased after P8. **b** Cell cycle profile of a representative sample depicting a G1 population, a proliferating S/G2/M population, and an apoptotic sub G1 population. **c**, **d** Analysis of five UC-MSC samples indicated decreased proliferation at P10 compared to P4 (**c**) and an increased apoptotic cell frequency at P10 compared to the earlier passages (**d**). **e** TF expression remained stable in the analyzed passages as indicated by flow cytometry. **f**–**g** Gene expression of the procoagulant factors TFF3 and COL1A1 (**f**) and the anticoagulant factors TFPI and PTGIR (**g**) was comparable in UC-MSCs over passages, except PTGIR tended to be decreased at higher passages compared to those in P2. **h** The PDT was analyzed for DP-MSCs, which demonstrated a lower growth rate at P7 and P8. **i** DP-MSCs tended to express a lower level of TF at higher passages. **j, k** Gene expression analysis of the procoagulant factors TFF3 and COL1A1 (**j**) and the anticoagulant factors TFPI and PTGIR (**k**) in DP-MSCs suggested comparable levels of these genes from P2 to P8

For advanced analysis, UC- and DP-MSCs were selected to represent MSC sources with high and moderate TF expression, respectively, unless otherwise indicated. To study how the TF level changes in correlation with MSC proliferation capacity and increasing passages, cells were cultured from P3 to P10, and the population doubling time (PDT) was calculated for every passage. The PDTs of UC-MSCs at P9 and P10 (mean ± SD) were 38.18 ± 6.31 and 39.22 ± 7.36, respectively, compared to those at P3 to P8, which ranged from 22.46 ± 2.82 to 26.80 ± 1.85. This suggests that UC-MSCs display a decreased growth capacity after P8 (P9 and P10 versus P3-6: *p* < 0.01, vs. P7: *p* < 0.05) (Fig. 3a). This observation correlates with data from the cell cycle analysis. A representative DNA profile of a UC-MSC sample is depicted in Fig. 3b, showing an apoptotic sub-G1 subset, a cell population in the G1 phase, and proliferating cells in S and G2. The proliferating fraction decreased over passages (P4 vs. P10: *p* < 0.01) (Fig. 3c), and apoptotic cells increased (P4 and P6 versus P10: *p* < 0.01) (Fig. 3d). On the other hand, their TF expression remained comparable from P2 to P10 (Fig. 3e). Quantitative PCR also revealed a stable expression level of the coagulant factors *TFF3* and *COL1A1* and the anticoagulant factor *TFPI* in the analyzed passages, while *PTGIR* tended to be decreasingly expressed at more advanced passages (Fig. 3f-k).

In the case of DP-MSCs, PDT increased over passages from 36.38 ± 7.46 at P3 to 89.18 ± 40.46 at P8 (P7 and P8 versus P3: *p* < 0.01 and 0.05, respectively) (Fig. 3h). The TF expression of DP-MSCs tended to decrease with increasing passages from P2 to P6 and increased again at P8, as indicated by flow cytometry (frequency of TF^+^ cells at P2 versus the one at P6: *p* < 0.05) (Fig. 3i) and quantitative real-time PCR (Fig. 3j). The expression of the other pro- and anticoagulant genes, including *COL1A1, TFPI*, and *PTGIR,* remained unchanged within the analyzed passages (Fig. 3j and k).

### Analysis of TF expression in MSCs depending on culture conditions

**Fig. 4:**
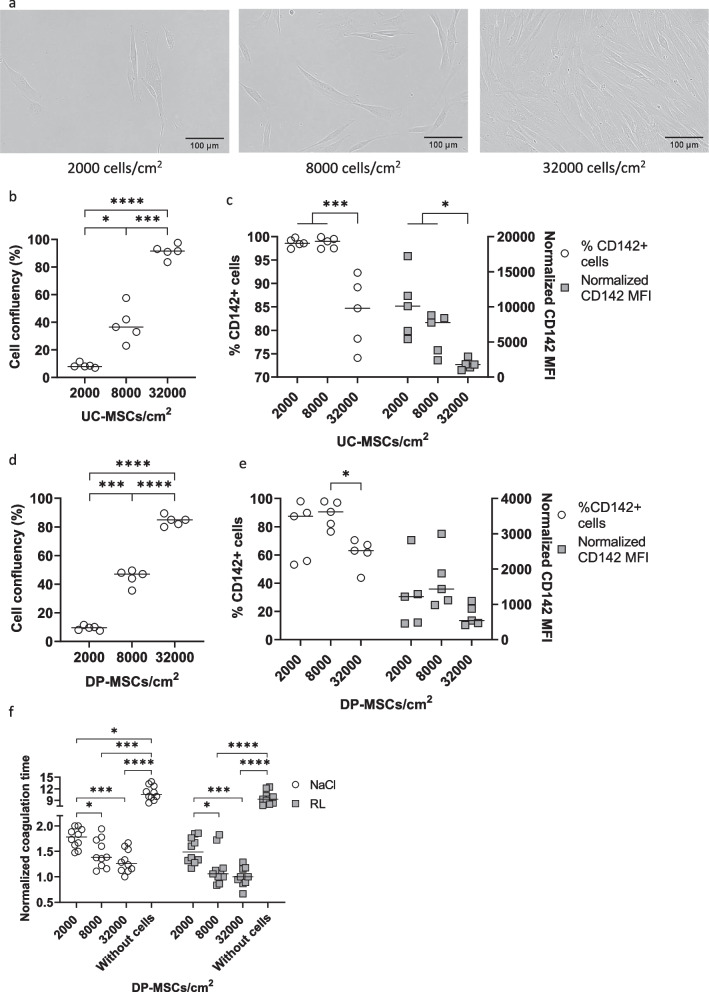
Impact of cell density on TF expression in UC- and DP-MSCs. **a** The cell morphology of UC-MSCs seeded at different densities is depicted. **b** The cell densities represent confluency between 8 ± 1.71 and 91.5 ± 5.05. **c** TF expression decreased when UC-MSCs became confluent. **d–e** Similar to UC-MSCs, cells derived from DP-MSCs were observed from 9.5 ± 1.61 to 85 ± 3.60 confluency (**d**), and TF expression was lower in confluent cells (**e**). **f** Despite their lower TF level, DP-MSCs at higher cell density increased their coagulant activity

To study how cell density might influence the TF expression of MSCs, UC-MSCs (Fig. 4a-c), DP-MSCs (Fig. 4d-f), and AT-MSCs (Additional file 3: Fig. S2b and S2c) were seeded at cell densities of 2000, 8000, and 32,000 cells/cm^2^. The cell morphology of a representative sample is depicted (Fig. 4a). The confluency of each density was measured (Fig. 4b, d and Additional file 3: S2a), and TF expression was analyzed by flow cytometry (Fig. 4c, 4e, and Additional file 3: S2b). The frequency of TF^+^ cells and TF MFI of UC- and DP-MSCs decreased when cells reached confluence (*p* < 0.05) (Fig. 4c and e). Similarly, AT-MSCs expressed less TF at higher cell densities (Additional file 3: Fig. S2c). Although TF expression decreased in confluent cells, these cells showed significantly higher coagulant activity than cells of lower density (Fig. 4f).

**Fig. 5:**
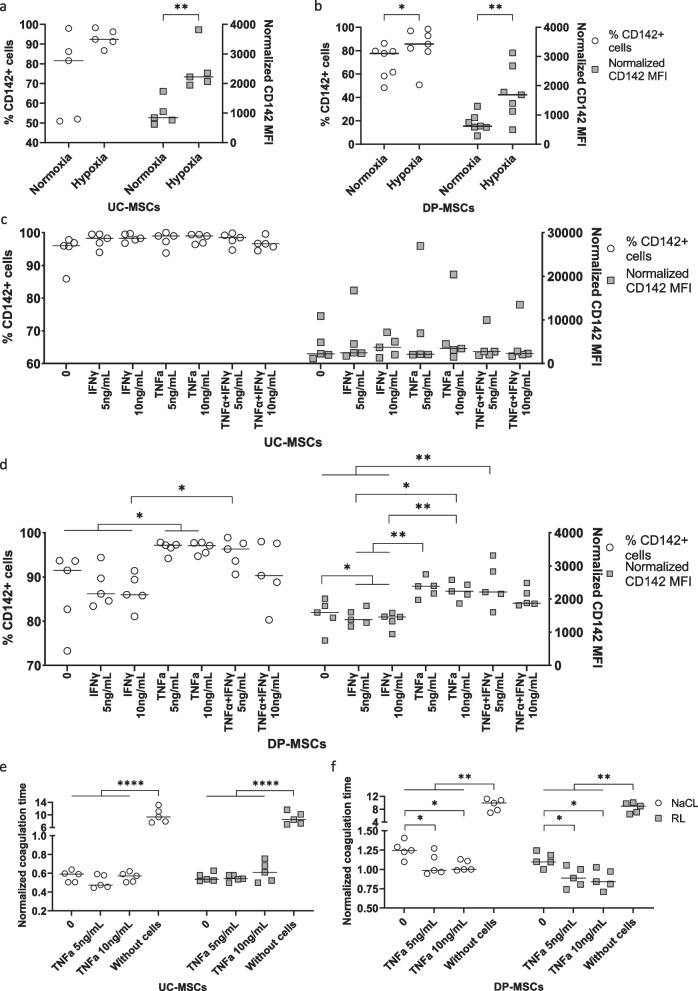
Impact of environmental factors on TF expression in UC- and DP-MSCs. **a**, **b** Culture of UC-MSCs (**a**) and DP-MSCs (**b**) in 2% oxygen conditions (hypoxia) upregulated TF expression compared to an ambient oxygen concentration of 21% (normoxia). **c** The inflammatory cytokines IFNγ and TNFα did not change TF levels in UC-MSCs. **d** On the other hand, TF levels were enhanced in DP-MSCs in the presence of TNFα but not IFNγ. **e** The coagulant activity of UC-MSCs did not change upon TNFα treatment. **f** The coagulant activity of DP-MSCs was higher upon TNFα treatment

As different environmental factors might affect TF expression, UC-MSCs were cultured under hypoxia (2% oxygen) compared to normoxia (21% oxygen) as well as in the presence of inflammatory cytokines, and TF expression was measured by flow cytometry. Both UC- and DP-MSCs exhibited higher TF under hypoxia than under normoxia (MFI: *p* < 0.01) (Fig. 5a and b). When the cells were primed with TNFα and IFNγ, TF expression on UC-MSCs did not change (Fig. 5c). On the other hand, TNFα and IFNγ affected TF expression on DP-MSCs differently. While TNFα tended to boost its level, IFNγ seemed to have the opposite effect (Fig. 5d). Accordingly, the coagulation time of UC-MSCs was comparable between the conditions (Fig. 5e), while that of DP-MSCs decreased in the presence of TNFα compared to the untreated samples (*p* < 0.05 in NaCl and *p* < 0.01 in Ringer’s lactate) (Fig. 5f). AT-MSCs behaved similarly to UC-MSCs, with no change in TF expression (Additional file 3: Fig. S2d) or coagulation time in the presence of TNFα (Additional file 3: Fig. S2e).

### Analysis of TF expression in MSCs depending on cell product preparation and storage

**Fig. 6:**
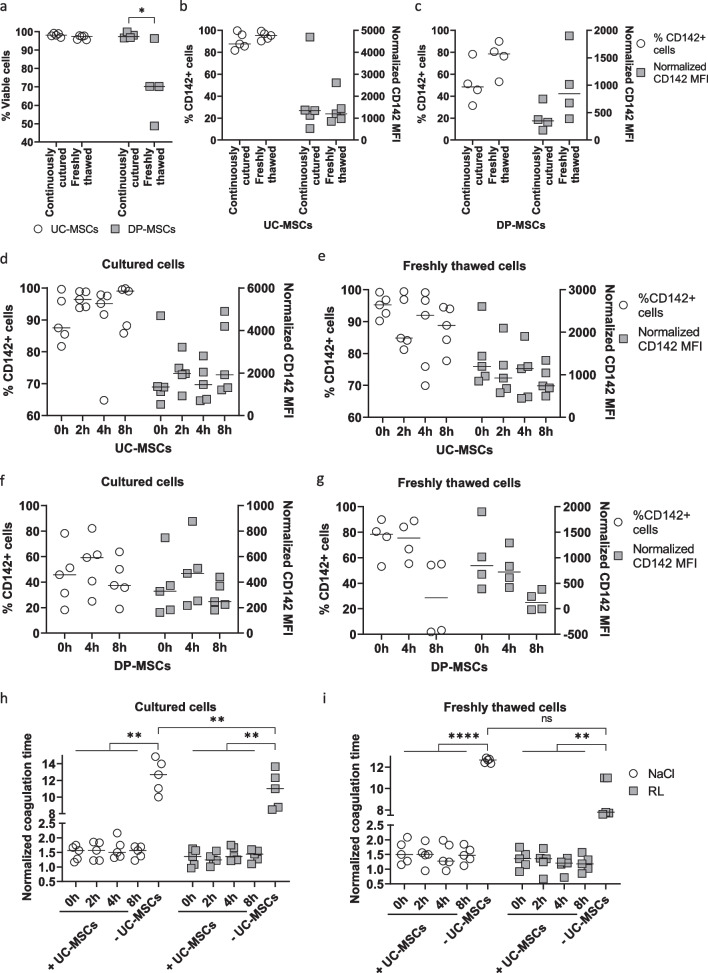
Impact of cell product preparation and storage on TF expression in UC- and DP-MSCs. **a**–**c** Both continuously cultured and cryopreserved/freshly thawed MSCs can be used for therapeutic purposes. Cell viability (**a**) and TF expression (**b**, **c**) were analyzed by flow cytometry. UC-MSCs (**b**) and DP-MSCs (**c**) under both conditions showed similar TF levels. **d–g ** The TF expression of continuously cultured and freshly thawed UC-MSCs (**d** and **e**, respectively) and DP-MSCs (**f** and **g**, respectively) was observed for up to 8 h of storage in NaCl. While TF was preserved on UC-MSCs of both conditions and continuously cultured DP-MSCs over the 8-h observation period, freshly thawed DP-MSCs tended to reduce its expression after eight hours. **h**, **i** The coagulation activity of continuously cultured (**h**) and cryopreserved/freshly thawed UC-MSCs (**i**) remained unchanged during an 8-h storage period

Both continuously cultured and cryopreserved/freshly thawed MSCs have been used as therapeutic products. Therefore, UC- and DP-MSCs prepared under these conditions were tested for cell viability and TF expression. While the viability of continuously cultured and cryopreserved/freshly thawed UC-MSCs was similar, cryopreserved/freshly thawed DP-MSCs were significantly less viable than their continuously cultured counterparts (Fig. 6a). TF expression was comparable in the analyzed conditions (Fig. 6b and c).

Furthermore, continuously cultured and cryopreserved/freshly thawed UC-, DP-, and AT-MSCs were stored in NaCl (Fig. 6d–g and Additional file 3: S2f) and Ringer’s lactate (Additional file 4: Fig. S3c–f) for up to eight hours to test their viability and TF expression during short-term storage. The viability of MSCs did not change during the 8-h storage (Additional file 4: Fig. S3a, b). TF was measured every two hours by flow cytometry, indicating that the storage of both continuously cultured and freshly thawed UC-MSCs and continuously cultured DP-MSCs in NaCl did not change their expression profile (Fig. 6d-f, respectively). In contrast, the TF levels of freshly thawed DP-MSCs and continuously cultured AT-MSCs dropped after 8 h of storage (Fig. 6g and Additional file 3: S2f). Similar results were observed when the cells were stored in Ringer’s lactate (Additional file 4: Fig. S3c-f). The in vitro clotting time of continuously cultured and cryopreserved/freshly thawed UC-MSCs was comparable within 8 h of storage in NaCl and Ringer’s lactate (Fig. 6h and i, respectively).

### Effects of allogenic UC-MSC administration on patients’ coagulation tests

**Fig. 7:**
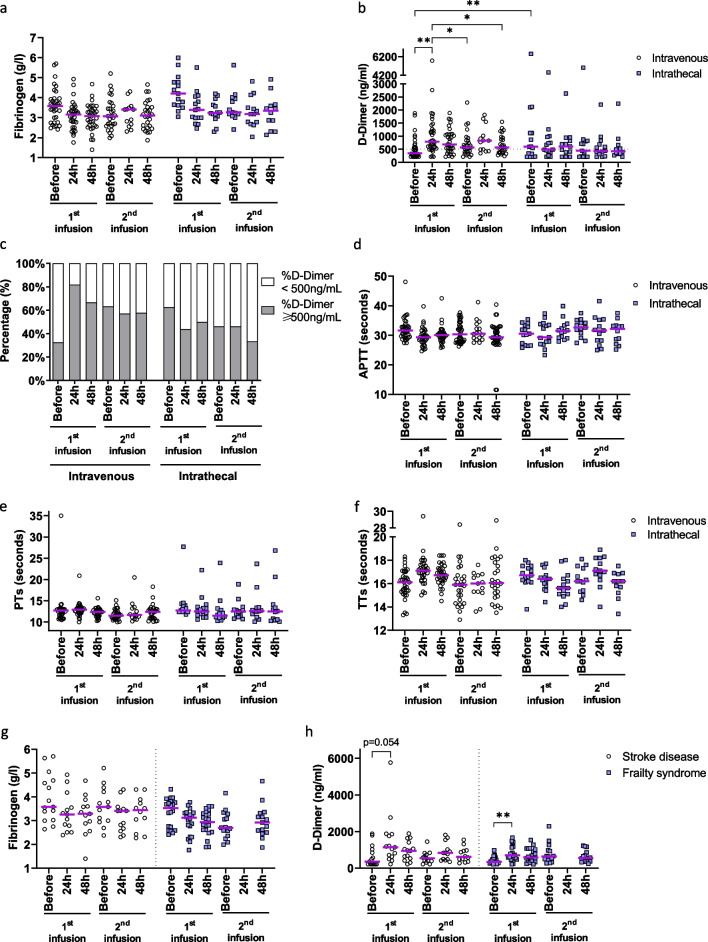
Coagulation induced by UC-MSCs upon administration in patients. **a** Patients were infused twice with UC-MSCs via intravenous or intrathecal routes. The fibrinogen concentration in blood was recorded at baseline and 24 and 48 h after the intervention. The fibrinogen level was comparable between the different analyzed time points. **b** D-dimer increased after 24 h and decreased again after 48 h in patients with systemic cell administration, while no significant change was observed in the intrathecal group. **c** Patients were classified into two groups with normal (< 500 ng/ml) and elevated D-dimer levels (≥ 500 ng/ml). Compared to the baseline, the numbers of intravenously injected patients with elevated D-dimer increased at 24 h and decreased again at 48 h after the first cell infusion, but this effect was not observed at the second cell infusion after 3 months. In the intrathecal group, the proportion of patients with elevated D-dimer remained comparable or even lower after both cell infusions. **d**–**f** There were no significant changes in aPPTs (**d**), PTs (**e**), or TTs (**f**) between the analyzed time points. **g**, **h** Patients receiving intravenous UC-MSCs were classified according to their background, including stroke and frailty syndromes. The fibrinogen (**g**) and D-dimer (**h**) concentrations of both groups showed similar tendencies

Coagulation was analyzed in patient serum before cell therapy and at 24 and 48 h after each MSC infusion. Patients diagnosed with stroke (*n* = 32) and frailty syndromes (*n* = 19) received allogenic UC-MSCs via either systemic infusion or local injection into the intrathecal space. To study the impact of the administration route on MSC-induced coagulation, we compared fibrinogen, D-dimer, aPTT, PT, and TT of these two patient groups. While fibrinogen and D-dimer are the substrate and the degraded product of the fibrin-forming reaction, the aPPT test measures the activity of the intrinsic and common pathways, the PT test evaluates the extrinsic and common pathways, and the TT test assesses the common pathway activity. There were no significant changes in the fibrinogen concentration in the serum of either patient group (Fig. 7a). Importantly, the D-dimer concentration was enhanced in patient serum at 24 h after the intravenous infusion of UC-MSCs and decreased again after 48 h. Its level was also elevated in the second injection; however, the effect was milder than that of the first injection (Fig. 7b). On the other hand, the intrathecal route showed comparable D-dimer levels at all analyzed time points (Fig. 7b). Accordingly, the proportion of patients with a D-dimer level higher than 500 µg/L, which is used to classify patients with a higher risk of venous thromboembolism [31], increased from 32.50% at baseline to 81.82% and 66.67% at 24 and 48 h after the first intravenous infusion, respectively (Fig. 7c). In the intrathecal group, 62.50%, 43.75%, and 50.00% of patients showed elevated D-dimer levels at the baseline, 24-h, and 48-h time points, respectively (Fig. 7c). Despite the elevated D-dimer levels, no patients showed syndromes of thrombosis. The coagulation tests revealed comparable aPTTs, PTs, and TTs at all analyzed time points in both the intravenous and intrathecal groups (Fig. 7d–f, respectively).

Furthermore, multiple linear regression was performed to analyze a potential correlation of the coagulation values with other clinical variables, including sex, age, and disease background. Serum fibrinogen depended on the disease background but not on the other parameters (Additional file 1: Table S1). There was no relationship between D-dimer, aPTT, PT, and TT and the analyzed variables (Additional file 1: Tables S2–S5, respectively). The fibrinogen and D-dimer levels of intravenously infused patients with stroke and frailty syndromes showed similar tendencies (Fig. 7g and h, respectively).

### Correlation between TF expression and coagulation in patients after UC-MSC administration

**Fig. 8:**
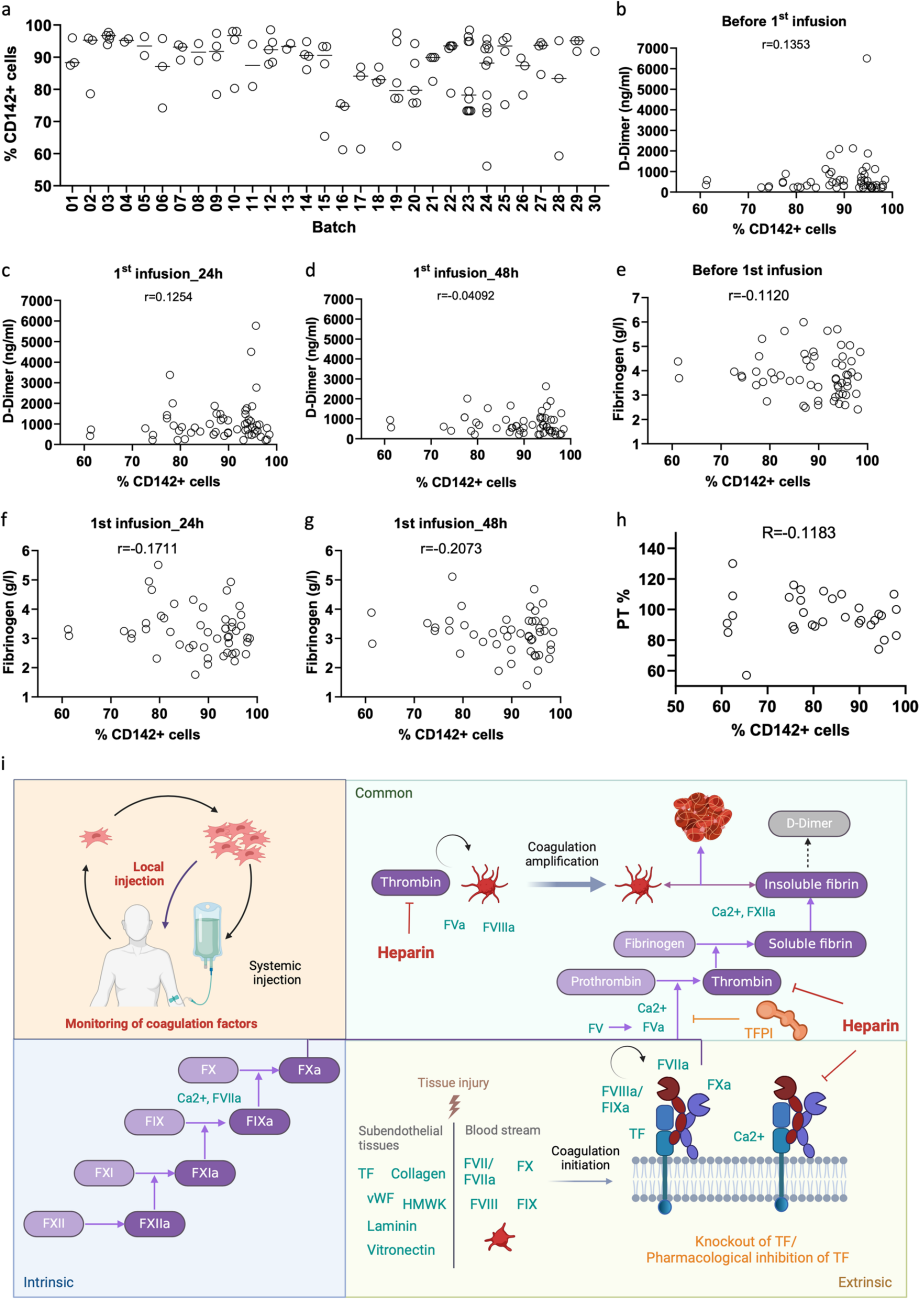
Correlation between TF expression and D-dimer levels in patients after UC-MSC administration. **a** TF expression was analyzed in 30 different UC-MSC batches. **b-d.** D-dimer levels in patient serum before cell therapy (**b**) and at 24 h (**c**) and 48 h (**d**) after the first UC-MSC infusion showed no correlation with the frequency of TF-expressing cells. **e**–**g** Fibrinogen levels in patient serum were comparable between the indicated analyzed time points. **h** Next, PT tests of patient serum were examined in the presence of UC-MSCs, showing a normal range of PT. Furthermore, the PT values were independent of TF expression on UC-MSCs. **I** The plot depicts the extrinsic (yellow box), intrinsic (blue box), and common (green box) coagulation cascades. TF, the initiator of the extrinsic pathway, is activated during blood vessel damage or in the presence of external factors such as bacterial endotoxin, inflammatory cytokines, and thrombin. During the initiation phase of coagulation, procoagulant factors such as TF, collagen, *von Willebrand factor* (vWF), laminin, vitronectin, and high molecular weight kininogen (HMWK) in subendothelial tissues are exposed to platelets and circulating coagulant factors FVII, FVIII, FIX, FX, etc. An extrinsic coagulation cascade is intermediately initiated. TF is decrypted, leading to proteolytic activation of its ligands FVII and FX into FVIIa and FXa. FXa cleaves prothrombin in thrombin. Thrombin proteolyzes fibrinogen into fibrin to form clots. Thrombin further activates platelets, FVa, and FVIIIa via a positive feedback loop to propagate the coagulation cascade. When clots are dissolved in the body, fibrin is broken into small protein fragments known as D-dimers. Genetic downregulation or pharmacological inhibition of TF could reduce TF-induced extrinsic coagulation. On the other hand, the data suggest that other coagulant factors are also involved in this process upon UC-MSC administration. Therefore, prophylactic treatment with anticoagulant drugs such as heparin is of benefit, as the drug also targets the common coagulant pathway. Furthermore, local administration might reduce the risk of thrombogenic events compared to systemic infusion

Because TF mediates the activation of FVII and FX in the coagulation cascade, the coagulant activity of patient serum was studied in relation to the TF expression of infused cells. TF expression in each manufacturing batch was analyzed by flow cytometry, showing a median frequency of TF^+^ cells (range): 90.30 (56.10–98.50) (Fig. 8a). There was no correlation between TF^+^ cell frequency and D-dimer at baseline or at 24 and 48 h after the first UC-MSC infusion (Fig. 8b–d, respectively). Fibrinogen was also not correlated with TF^+^ cell frequency (Fig. 8e–g). Furthermore, patient serum was collected at baseline to perform the PT test in the presence of 2500 UC-MSCs, which corresponded to approximately 2.10^6^ per kg infused cells in an adult estimated with 60 kg body weight and 5 L blood. UC-MSCs expressed diverse frequencies of TF ranging from 61.20 to 98.00. However, PT did not show a correlation with TF expression (Fig. 8h).

## Discussion

To date, data on TF expression in xeno- and serum-free cultured MSCs remain elusive. Here, we report for the first time the TF expression of MSCs derived from AT, BM, DP, and UC that were expanded in a xeno- and serum-free StemMACS MSC expansion media kit XF. AT- and UC-MSCs expressed the highest TF protein levels, followed by DP-MSCs and BM-MSCs, while HUVECs were negative for TF. Both BM-MSCs and DP-MSCs express a wide range of TFs among different donors. This was in line with previous studies on FBS-cultured MSCs, in which MSCs derived from AT, UC, and the amniotic membrane showed superior TF expression compared to BM-MSCs [27, 32].

TF expression can be influenced by many factors both in vitro and in vivo*.* Endotoxin, reactive protein C, and inflammatory cytokines such as IL-6, IL-1β, and TNF-α induce strong upregulation of TF in endothelial cells, monocytes/macrophages, and vascular smooth muscle cells [33, 34], while anti-inflammatory cytokines have the opposite effect [35, 36]. Growth factors and serum also trigger TF generation [37, 38]. This suggests that culture conditions might influence the expression levels of TF in MSCs. Indeed, UC-MSCs cultured in human platelet lysate increased their TF level compared to those expanded in FBS-containing media [27]. Moreover, AT-MSCs at advanced passages showed reduced TF expression compared to their younger counterparts, while BM-MSCs showed high variations in this factor between donors without significant changes over passages [28]. Others observed a tendency of increased TF expression in BM-MSCs at passage 6 compared with that at passage 3 and even higher expression in cells stimulated with activated PBMNCs [39]. In our xeno- and serum-free culture platform, UC- and DP-MSCs retained stable TF expression independent of their proliferation state. Furthermore, UC- and AT-MSCs showed similar TF surface levels in the presence of TNFα and IFNγ, while DP-MSCs increased TF expression upon TNFα activation. The results suggest that MSCs differ from endothelial cells and monocytes/macrophages in the regulation of TF expression. This could be related to the high level of TF on MSCs compared to these cell types, which only express TF upon stimulation [33, 34]. Due to their immunomodulatory potency, MSCs are used to treat immune-activated conditions such as graft-versus-host disease after hematopoietic stem cell transplantation, cytokine release syndrome during SARS-CoV-2 infection, and asthma [40–42]. Our data suggest that MSCs might change their TF expression depending on their nature and the inflammatory cytokines to which they are exposed. Moreover, culture of UC- and DP-MSCs under hypoxia with 2% oxygen was associated with higher TF expression. In line with our observations, hypoxia was shown to augment TF expression in ischemia-associated thrombosis [43, 44] and cancer [45–47]. This could have a major impact on MSC therapy, since the majority of therapeutic MSC products are cultured in atmospheric oxygen conditions and the cells must adapt to physiological oxygen concentrations ranging from 1 to 9% [48, 49]. MSCs may upregulate TF expression in vivo and therefore increase the risk of thrombosis in patients.

In addition to transcriptional regulation of the TFF3 gene, the procoagulant activity of the TF/FVIIa/FXa complex is dependent on its conformation and posttranslational modifications [50]. Phospholipids, including PS and phosphatidylethanolamine, transform TF into a decrypted (active) form, while sphingomyelin preserves its encrypted state [50, 51]. Because the plasma membrane is asymmetrically distributed, PS and phosphatidylethanolamine are exposed to the cytosolic site, while sphingomyelin is exhibited on the cell surface [52, 53]. As a result, the proteolytic activity of the TF/FVIIa/FXa complex remains low in intact cells [54, 55]. Once cells are damaged or activated, TF is exposed to PS and phosphatidylethanolamine, leading to the formation of Cys^186^–Cys^209^ bisulfide binding in the extracellular domain of TF and consequently transforming the TF/FVIIa/FXa complex into a highly active state [55, 56]. Once MSCs are infused into patients, they might interact with several in vivo coagulant factors, such as Ca^2+^ ions, adenosine triphosphate, or reactive oxygen species. These factors are known to be involved in TF decryption and the release of TF^+^ microvesicles, leading to pathologic thrombus formation [54, 57, 58]. The authors observed increased D-dimer levels in patient serum at 24 h after intravenous UC-MSC infusions, suggesting activation of the coagulation cascade. Because thrombin, a key player in the coagulation cascade, affects TF expression through a positive feedback loop, the cascade is capable of self-propagation once initiated [59]. However, D-dimer decreased at 48 h after the treatment. No patients developed thrombosis, suggesting that prophylactic treatment with the anticoagulant Lovenox 4000 Ui or Xarelto 10 mg successfully prevented amplified coagulation reactions. Patients with intrathecal UC-MSC infusions showed no abnormal coagulation values, supporting a lower risk of local administration to develop in vivo thrombosis.

MSC-induced coagulation is thought to be mediated through exposure of TF molecules on the cell surface to blood [28]. Previous studies demonstrated that TF expression on MSCs was correlated with the activation of instant blood-mediated inflammatory reactions [28, 39]. Treatment of MSCs with an FVIIa inhibitor or inhibitory anti-TF-antibody 4509 suppressed blood clotting by interfering with the TF-FVIIa-FXa complex of the extrinsic coagulation pathway [39]. Therefore, TF was thought to be the main trigger of MSC-induced blood clotting. However, the authors observed no correlation between TF expression and D-dimer serum levels after UC-MSC administration. Moreover, patient serum was tested for PT values in the presence of UC-MSCs, showing comparable PT values between samples with different frequencies of TF-expressing cells. These data suggest that other factors of the coagulation and fibrinolysis system are also involved in this process. Indeed, TF-negative HUVECs and DP-MSCs with as low as 0.2% TF-positive cells were able to induce coagulation in vitro*,* as demonstrated by our and others’ data [60].

Proteomic analysis of MSCs using mass spectrometry revealed an enriched expression of platelet activation factors in MSCs from adipose, BM, placenta, and Wharton’s-jelly-derived MSCs [61]. Additional file 1: Table S6 summarizes factors of the coagulant and fibrinolytic systems. MSCs exhibited reasonable levels of PS on their surface, as approximately 10% of UC-MSCs in culture and 8% of DP-MSCs bound to annexin V. Cryopreserved and thawed UC- and DP-MSCs exhibited slightly higher frequencies of annexin V^+^ cells. Silachev et al*.* reported that 4% of MSCs exposed PS and inhibition of PS by preincubation with Annexin V interfered with their coagulant properties [14]. Araldi et al*.* observed that more than 50% of DP-MSCs were PS positive [60]. PS stimulates coagulation via TF-dependent and TF-independent mechanisms. During the initiation phase, it can transform inactive and encrypt TF into decrypt form, which allows full activation of the TF/FVIIa/FXa complex [50, 51]. Furthermore, PS also promotes the binding of coagulation factors (VIIa, IXa, Xa and IIa) to the membrane, allowing them to interact and activate each other [62]. Another candidate of interest is the antifibrinolytic plasminogen activator inhibitor-1 (PAI-1). Many cells, such as endothelial cells, megakaryocytes and leukocytes, smooth muscle cells, fibroblasts, adipocytes, and hepatocytes, secrete PAI-1 into the blood stream and subendothelial space [63]. It acts as an inhibitor of plasminogen activators to attenuate fibrin degradation. Increased levels of PAI-1 have been reported to be associated with thrombotic pathologic conditions, including cardiovascular diseases as well as cancer aggressiveness [64, 65]. MSCs can increase PAI-1 levels in the blood stream via both direct secretion of this factor and an indirect mechanism, in which their exosomes induce PAI-1 expression in vascular smooth muscle cells and vascular endothelial cells [66, 67]. D-dimer, a fibrin degradation product, was increased in patient serum upon UC-MSC infusion, suggesting that fibrinolysis occurred during the short-term analysis period. Further studies might investigate the mechanism and long-term effects of PAI-1 on the procoagulant activity of MSCs.

Emerging evidence supports the important role of lipid mediators in the immunomodulatory and regenerative properties of MSCs [68]. In experimental acute lung injury, a lipid mediator named lipoxin A4 (LXA4) promoted tissue repair, reduced inflammation, and prolonged the lifespan of BM-derived MSC-treated mice [69]. LXA4 interacts with its receptor, ALX/FPR2, leading to the downregulation of TGF-ß/Smad signaling [70]. As a result, MSCs reduced fibrosis in renal tissue, improved renal function, and extended survival in a mouse model of diabetic nephropathy. Although the interaction of LXA4 with immune cells and its effects on immunomodulation have been intensively studied, the coagulant activity of MSCs at different LXA4 production levels remains elusive. Hence, studies should be performed to examine a potential link between MSC-produced lipid mediators and coagulation.

Recently, TF and hemocompatibility assessments were recommended for inclusion in the minimal criteria of MSC products [71]. Depleting the TF^+^ BM-MSC population ameliorated the thrombogenic activity of infused cells while maintaining their immunomodulation [27]. A recent study selected only DP-MSC lots with less than 25% TF + cells to mitigate thrombogenesis in critically ill COVID-19 patients. Cell therapy did not elevate thrombogenic risk in this patient group when Lovenox was administered as supportive therapy [60]. This study also demonstrated that rotation thromboelastometry (ROTEM) was insufficient to evaluate the clotting risk of MSCs, as it increased their exposure to PS, which mediates the coagulation pathway independently from TF. Importantly, this approach is limited to only MSCs with low to moderate TF expression and is not suitable for highly TF-expressing cells such as UC- and AT-MSCs. Furthermore, although the assay is suitable to quantify the coagulation property of cell therapeutic products, it might fail to reflect their behaviors in vivo*,* as many environmental milieus, such as inflammation, hypoxia, or Ca2^+^ in serum, might immensely change their TF-mediated coagulant activity. Anticoagulant drugs such as heparin would be a better choice, as they have successfully prevented thrombogenic events after intravenous infusions of several MSC types, including BM-, DP-, and UC-MSCs, as shown here [27, 60]. However, some patients developed high D-dimer levels after therapy despite this prophylactic treatment and needed closer monitoring, in which coagulation laboratory tests should be regularly performed and anticoagulant drugs can be prescribed if needed (Fig. 8k).

## Conclusions

Overall, our study acknowledges the relevance of the choice of MSC types and administration routes to reduce hypercoagulant risk in patients upon MSC infusions. We also demonstrated that the prophylactic use of anticoagulant drugs and monitoring of coagulation indicators were sufficient to prevent thrombosis caused by UC-MSCs, which expressed the highest level of procoagulant factors. Furthermore, our data suggested that in vitro analysis of TF expression, prophylactic use of TF inhibitors or selective administration of cell products with low TF expression might be less effective for this purpose. Overall, the study addresses a fundamental aspect of MSC-based therapy regarding its safety profile and provides important hints to develop reasonable strategies against its thrombogenic side effects.

### Supplementary Information

**Additional file 1:** Supplementary tables descibe linear regression analysis of clinical parameters of patients infused with UC-MSCs and a summary of coagulant and fibrinolytic factors.

**Additional file 2: Figure S1.** Analysis of the in vitro MSC-induced clotting and levels of phosphatidylserine (PS) and TF on MSCs.** a** Comparative study of the coagulant activity of MSCs derived from AT, BM, DP and UC.** b** Detection of Annexin V binding PS on the surface of DP- and UC-MSCs by flow cytometry.** c** and** d** Expression of TF in the Annexin V+7-AAD-early apoptotic and Annexin V+7-AAD+ late apoptotic cell populations.

**Additional file 3: Figure S2.** TF expression and activity on AT-MSCs.** a** Treatment of AT-MSCs with the anti-TF inhibitory antibody (clone HTF-1) resulted in a partial reduction in their coagulant activity.** b**-**f** Impact of culture conditions on TF expression and activity in AT-MSCs.** b** AT-MSCs were seeded at three different concentrations, and their cell density was measured.** c** The corresponding TF expression levels were analyzed, showing lower TF expression at the highest cell density.** d** AT-MSCs did not change TF expression in the presence of the inflammatory cytokines INFg and TNFa.** e** Furthermore, both AT-MSCs and their TNFa-treated counterparts indicated comparable coagulation activity when incubated with healthy plasma.** f** Storage of AT-MSCs might reduce TF levels.

**Additional file 4: Figure S3.** Impact of storage on the survival and TF expression of UC- and DP-MSCs.** a** and** b** The survival rate of UC-MSCs (**a**) and DP-MSCs (**b**) was observed for up to 8 h storage in NaCl.** c**–**f** TF expression of cultured UC-MSCs (**c**), freshly thawed UC-MSCs (**d**), cultured DP-MSCs (**e**), and freshly thawed UC-MSCs (**f**) within 8 h of storage in RL is depicted.

## Data Availability

Clinical protocols are available at www.clinicaltrials.gov, no. NCT05292625 and NCT04919135. The datasets used and/or analyzed during the current study are available from the corresponding author upon reasonable request.

## Abbreviations

aPTT: Activated partial thromboplastin time
AT: Adipose tissue
FVII: Coagulation factor VII
FX: Coagulation factor X
COL1A1: Collagen 1A1
DP: Dental pulp
GAPDH: Glyceraldehyde-3-phosphate dehydrogenase
HUVECs: Human umbilical vein endothelial cells
IFN: Interferon
MFI: Median fluorescence intensity
MSC: Mesenchymal stem/stromal cell
P: Passage
PBMNCs: Peripheral blood mononuclear cells
PDT: Population doubling time
PS: Phosphatidylserine
PT: Prothrombin time
PTGIR: Prostaglandin I2 receptor
RL: Ringer lactate
TT: Thrombin time
TF: Tissue factor
TFPI: TF protein inhibitor
TNF: Tumor necrosis factor
UC: Umbilical cord
