# Supplementary material for: Impact of tissue factor expression and administration routes on thrombosis development induced by mesenchymal stem/stromal cell infusions: re-evaluating the dogma
Source: Stem Cell Res Ther. 2024 Feb 27;15:56. doi: 10.1186/s13287-023-03582-3 (PMC10900728; doi:10.1186/s13287-023-03582-3)
Supplement: Supplementary file 1 — Additional file 1: Supplementary tables descibe linear regression analysis of clinical parameters of patients infused with UC-MSCs and a summary of coagulant and fibrinolytic factors. [file 13287_2023_3582_MOESM1_ESM.docx]

**Supplementary tables**

**Impact of tissue factor expression and administration routes on thrombosis development induced by mesenchymal stem/stromal cell infusions: re-evaluating the dogma**

Van T. Hoang^1*#^, Duc Son Le^1#^, Duc M. Hoang^1^, Trang Thi Kieu Phan^1^, Lan Anh Thi Ngo^2^, Trung Kien Nguyen^1^, Bui Viet Anh^2^, Liem Nguyen Thanh^1,3,4*#^

Table S1: Linear regression analysis to analyze a potential correlation between fibrinogen and clinical parameters, including sex, age, disease background, and administration route, at each analyzed time point.

| **Variable** | **Fibrinogen (g/L)** | | | | | | | | | | | |
| --- | --- | --- | --- | --- | --- | --- | --- | --- | --- | --- | --- | --- |
|  | *Baseline*  *(n=50)* | | *24 hours after the 1^st^ infusion (n=49)* | | *48 hours after the 1^st^ infusion (n=48)* | | *3 months after the 1st and before the 2nd infusion*  *(n=41)* | | *24 hours after the 2^nd^ infusion (n=26)* | | *48 hours after the 2^nd^ infusion (n=39)* | |
|  | β | 95% CI | β | 95% CI | β | 95% CI | β | 95% CI | β | 95% CI | β | 95% CI |
| **Gender**  Male (Ref)  Female | -0.13 | (-0.62 – 0.37) | 0.06 | (-0.36 – 0.47) | 0.30 | (-0.08 – 0.68) | 0.18 | (-0.32 – 0.68) | 0.21 | (-0.43 – 0.84) | 0.54* | (0.05 – 1.03) |
| **Age** | 0.02 | (-0.01 – 0.05) | 0.03* | (0.003 – 0.05) | 0.01 | (-0.01 – 0.03) | 0.01 | (-0.02 – 0.03) | 0.02 | (-0.01 – 0.05) | 0.02 | (-0.01 – 0.05) |
| **Type of diseases**  Frailty (Ref)  Stroke | 0.84* | (0.18 – 1.50) | 0.74* | (0.18 – 1.31) | 0.57* | (0.05 – 1.08) | 0.94* | (0.27 – 1.61) | NA | NA | 0.82* | (0.18 – 1.46) |
| **Route**  Intravenous (Ref)  Intrathecal | 0.26 | (-0.37 – 0.89) | -0.01 | (-0.56 – 0.54) | -0.02 | (-0.52 – 0.47) | -0.14 | (-0.76 – 0.48) | -0.05 | (-0.65 – 0.56) | -0.28 | (-0.87 – 0.31) |
|  | R^2^= 26%, p = 0.01* | | R^2^= 21%, p = 0.03* | | R^2^= 17%, p = 0.08 | | R^2^= 24%, p = 0.04* | | R^2^= 11%, p = 0.45 | | R^2^= 24%, p = 0.04* | |

** p < 0.05*

Table S2: Linear regression analysis to analyze a potential correlation between D-dimer and clinical parameters, including sex, age, disease background, and administration route, at each analyzed time point.

| **Variable** | **D-Dimer (μg/L)** | | | | | | | | | | | |
| --- | --- | --- | --- | --- | --- | --- | --- | --- | --- | --- | --- | --- |
|  | *Baseline*  *(n=51)* | | *24 hours after the 1^st^ infusion (n=51)* | | *48 hours after the 1^st^ infusion (n=48)* | | *3 months after the 1^st^ and before the 2^nd^ infusion*  *(n=41)* | | *24 hours after the 2^nd^ infusion (n=26)* | | *48 hours after the 2^nd^ infusion (n=39)* | |
|  | β | 95% CI | β | 95% CI | β | 95% CI | β | 95% CI | β | 95% CI | β | 95% CI |
| **Gender**  Male (Ref)  Female | -279.6 | (-841.1 – 282.0) | -308.6 | (-891.3 – 274.1) | -216.7 | (-524.3 – 91.0) | -240.1 | (-798.4 – 318.2) | -432.4 | (-869.6 – 4.8) | 4.6 | (-312.1 – 321.4) |
| **Age** | 4.4 | (-29.1 – 38.0) | 9.9 | (-24.0 – 43.8) | 6.6 | (-11.9 – 25.2) | -5.6 | (-37.2 – 26.0) | 18.8 | (-3.7 – 41.3) | 1.6 | (-16.3 – 19.6) |
| **Type of diseases**  Frailty (Ref)  Stroke | 176.4 | (-573.1 – 925.9) | 659.8 | (-116.0 – 1435.6) | 270.6 | (-140.7 – 682.0) | -362.3 | (-1115.8 – 391.1) | N.A | N.A | 122.0 | (-290.1 – 534.1) |
| **Route**  Intravenous (Ref)  Intrathecal | 531.3 | (-181.8 – 1244.4) | -610.2 | (-1348.7 – 128.2) | -241.0 | (-642.4 – 160.4) | 353.7 | (-336.3 – 1043.8) | -214.9 | (-630.7 – 201.0) | -138.5 | (-521.7 – 244.8) |
|  | R^2^= 12%, p = 0.20 | | R^2^= 11%, p = 0.22 | | R^2^= 10%, p = 0.32 | | R^2^= 4%, p = 0.79 | | R^2^= 26%, p = 0.07 | | R^2^= 1.8%, p = 0.95 | |

** p < 0.05*

Table S3: Linear regression analysis to analyze a potential correlation between the activated partial thromboplastin time (aPTT) and clinical parameters, including sex, age, disease background, and administration route, at each analyzed time point.

| **Variable** | **aPTT (min)** | | | | | | | | | | | |
| --- | --- | --- | --- | --- | --- | --- | --- | --- | --- | --- | --- | --- |
|  | *Baseline*  *(n=50)* | | *24 hours after the 1^st^ infusion (n=48)* | | *48 hours after the 1^st^ infusion (n=48)* | | *3 months after the 1^st^ and before the 2^nd^ infusion*  *(n=41)* | | *24 hours after the 2^nd^ infusion (n=26)* | | *48 hours after the 2^nd^ infusion (n=38)* | |
|  | β | 95% CI | β | 95% CI | β | 95% CI | β | 95% CI | β | 95% CI | β | 95% CI |
| **Gender**  Male (Ref)  Female | 0.14 | (-2.04 – 2.33) | -0.25 | (-2.39 – 1.88) | 0.64 | (-1.24 – 2.51) | -1.13 | (-3.03 – 0.77) | 2.53 | (-1.34 – 6.40) | -0.29 | (-3.56 – 2.99) |
| **Age** | -0.03 | (-0.17 – 0.11) | -0.02 | (-0.14 – 0.11) | -0.04 | (-0.15 – 0.07) | -0.08 | (-0.19 – 0.03) | -0.11 | (-0.32 – 0.08) | -0.02 | (-0.21 – 0.16) |
| **Type of diseases**  Frailty (Ref)  Stroke | 2.11 | (-0.81 – 5.02) | 1.05 | (-1.88 – 3.97) | 1.05 | (-1.53 – 3.62) | 3.11* | (0.55 – 5.67) | NA | NA | 2.32 | (-1.94 – 6.58) |
| **Route**  Intravenous (Ref)  Intrathecal | -3.01* | (-5.80 – -0.21) | 0.43 | (-2.37 – 3.22) | 0.84 | (-1.63 – 3.31) | -0.59 | (-2.93 – 1.76) | 0.69 | (-4.37 – 2.99) | -0.18 | (-4.14 – 3.78) |
|  | R^2^= 11%, p = 0.23 | | R^2^= 5%, p = 0.70 | | R^2^= 10%, p = 0.32 | | R^2^= 39%, p = 0.001* | | R^2^= 10%, p = 0.46 | | R^2^= 8%, p = 0.58 | |

** p < 0.05*

Table S4: Linear regression analysis to analyze a potential correlation between the prothrombin time (PT) and clinical parameters, including sex, age, disease background, and administration route, at each analyzed time point.

| **Variable** | **PT (min)** | | | | | | | | | | | |
| --- | --- | --- | --- | --- | --- | --- | --- | --- | --- | --- | --- | --- |
|  | *Baseline*  *(n=51)* | | *24 hours after the 1^st^ infusion (n=49)* | | *48 hours after the 1^st^ infusion (n=48)* | | *3 months after the 1^st^ and before the 2^nd^ infusion*  *(n=41)* | | *24 hours after the 2^nd^ infusion (n=26)* | | *48 hours after the 2^nd^ infusion (n=39)* | |
|  | β | 95% CI | β | 95% CI | β | 95% CI | β | 95% CI | β | 95% CI | β | 95% CI |
| **Gender**  Male (Ref)  Female | 1.71 | (-0.57 – 3.99) | 1.03 | (-0.17 – 2.23) | 0.74 | (-0.53 – 2.00) | 0.09 | (-1.01 – 1.20) | 2.76 | (0.03 – 5.48) | 1.98 | (-0.25 – 4.20) |
| **Age** | 0.04 | (-0.09 – 0.18) | 0.06 | (-0.01 – 0.13) | -0.002 | (-0.08 – 0.07) | -0.03 | (-0.09 – 0.03) | 0.03 | (-0.11 – 0.17) | -0.01 | (-0.14 – 0.11) |
| **Type of diseases**  Frailty (Ref)  Stroke | 2.77 | (-0.28 – 5.81) | 1.70* | (0.05 – 3.34) | 0.71 | (-1.03 – 2.44) | -0.47 | (-1.97 – 1.02) | NA | NA | 1.16 | (-1.73 – 4.06) |
| **Route**  Intravenous (Ref)  Intrathecal | -0.48 | (-3.38 – 2.42) | -0.64 | (-2.23 – 0.94) | -0.06 | (-1.72 – 1.61) | 1.27 | (-0.09 – 2.64) | 0.02 | (-2.57 – 2.61) | 0.57 | (-2.12 – 3.26) |
|  | R^2^= 11%, p = 0.26 | | R^2^= 15%, p = 0.12 | | R^2^= 5%, p = 0.70 | | R^2^= 12%, p = 0.30 | | R^2^= 22%, p = 0.14 | | R^2^= 13%, p = 0.29 | |

** p < 0.05*

Table S5: Linear regression analysis to analyze a potential correlation between thrombin time (TT) and clinical parameters, including sex, age, disease background, and administration route, at each analyzed time point.

| **Variable** | **TT (min)** | | | | | | | | | | | |
| --- | --- | --- | --- | --- | --- | --- | --- | --- | --- | --- | --- | --- |
|  | *Baseline*  *(n=50)* | | *24 hours after the 1^st^ infusion (n=49)* | | *48 hours after the 1^st^ infusion (n=48)* | | *3 months after the 1^st^ and before the 2^nd^ infusion*  *(n=41)* | | *24 hours after the 2^nd^ infusion (n=26)* | | *48 hours after the 2^nd^ infusion (n=38)* | |
|  | β | 95% CI | β | 95% CI | β | 95% CI | β | 95% CI | β | 95% CI | β | 95% CI |
| **Gender**  Male (Ref)  Female | 0.09 | (-0.60 – 0.78) | -0.27 | (-1.47 – 0.93) | -9.62 | (-21.79 – 2.55) | -0.23 | (-1.91 – 1.45) | -0.60 | (-1.72 – 0.53) | 0.44 | (-1.42 – 2.30) |
| **Age** | 0.04 | (-0.01 – 0.08) | 0.04 | (-0.03 – 0.11) | 0.61 | (-0.13 – 1.34) | 0.02 | (-0.08 – 0.11) | 0.03 | (-0.02 – 0.09) | -0.04 | (-0.14 – 0.07) |
| **Type of diseases**  Frailty (Ref)  Stroke | -0.02 | (-0.94 – 0.90) | 0.85 | (-0.79 – 2.49) | -3.31 | (-20.01 – 13.39) | -0.01 | (-2.27 – 2.25) | NA | NA | -1.50 | (-3.92 – 0.92) |
| **Route**  Intravenous (Ref)  Intrathecal | 0.79 | (-0.09 – 1.67) | -1.47 | (-3.05 – 0.11) | -1.69 | (-17.70 – 14.32) | 0.23 | (-1.84 – 2.30) | 1.33* | (0.26 – 2.40) | -0.03 | (-2.25 – 2.20) |
|  | R^2^= 15%, p = 0.12 | | R^2^=9%, p = 0.35 | | R^2^= 13%, p = 0.19 | | R^2^= 0.7%, p = 0.99 | | R^2^= 29%, p = 0.05* | | R^2^= 8%, p = 0.61 | |

** p < 0.05*

***Table S6: Coagulant and fibrinolytic factors.***

| **No.** | **Protein** | **Functions in hemostasis and coagulation** | **Expression** | **Ref** |
| --- | --- | --- | --- | --- |
|  | **Pro-coagulant factors** | | | |
| 1 | von Willebrand factor (vWF) | Maintening hemostasis through platelet adhesion and Factor VIII stabilization | Mainly expressed by vascular endothelial cells and megakaryocytes; expressed by some cancer cells | (1,2) |
| 2 | Laminin | A major component of the basal lamina in animal tissues; Promoting FXII-dependent coagulation | A large family with many isotypes; expressed by almost all cell types, but its isotype composition depending on tissue types | (3,4) |
| 3 | Thrombospondin | Calcium-binding glycoprotein supporting platelet aggregation | Released from the α-granules of aggregating platelets; thrombospondin 1 and 2 were expressed in umbilical cord blood derived MSCs and regulated their chondrogenic differentiation | (5,6) |
| 4 | Fibrinogen | Component of the comment coagulation cascade, converting into fibrin during coagulation. | Synthesized in the liver | (7) |
| 5 | Vitronectin | Plasma protein binding to activated platelets resulting in their enhanced aggregation; inhibition of fibrinolysis by mediating the interaction of type 1 plasminogen activator inhibitor with fibrin | Secreted by liver cells | (8,9) |
| 6 | High molecular weight kininogen (HMWK) | An initiator of the intrinsic coagulation cascade | Secreted by liver cells | (10) |
| 7 | Collagen | Interacting with platelet collagen receptors to mediate platelet adhesion and activation at the site of injury. | There are 28 different types of collagen. Among them, type I is the most abundant. It presents in skin, the vascular system, lung, liver, intestine, bone, and tendon, etc. Most cells of mesenchymal origins express collagen type 1, e.g. fibroblasts, osteoblasts, and odontoblasts etc. | (11–13) |
| 8 | Negatively charged phospholipid | During the initiation phase: Decrypting of TF to fully activate the TF/FVIIa/FXa complex; Promoting the binding of coagulation factors (VIIa, IXa, Xa and II) to the membranes allowing them to interact and activate each other.  During the propagation phase: Promoting the thrombin generation by accumulation of tenase and prothrombinase on the platelet surface. | Present on the cell surface of activated or apoptotic cells when the membrane asymmetry is disrupted by activation of the enzyme scramblase or inactivation of flippase and floppase | (14–16) |
|  | **Anti-coagulant factors** | | | |
| 9 | Antithrombin III | Inhibitor of clotting factors IIa (thrombin), Xa, and with lower affinity IXa and XIIa | Secreted by liver cells, endothelial cells | [(11,12)](https://doi.org/10.1016/B978-0-12-809657-4.99756-4) |
| 10 | Protein C | Regulator of the coagulation factors including FVIIIa and FVa to interfere with the coagulation cascade | Synthesized predominantly by the liver | (17,18) |
| 11 | Protein S | Inhibitor of FXa, FVa, and FIXa; Cofactor of other anticoagulants such as activated protein C and tissue factor pathway inhibitor | Secreted by endothelial cells, megakaryocytes, hepatocytes, and Leydig cells | (19,20) |
| 12 | Tissue factor pathway inhibitor (TFPI) | Binding to the active site and blocking TF-FVIIa-complex and prothrombinase TFPI to supress the blood coagulation pathway | Expressed by numerous cell type, e.g. the endothelial cells of the microvasculature, smooth muscle cells, monocytes/macrophages, megakaryocytes/platelets, mesangial cells, fibroblasts, microglia, cardiomyocytes, and mesothelial cells | (21) |
| 13 | Prostaglandin I2 Receptor (PTGIR) | A G protein-coupled receptor  of platelet membrane that inhibits platelet aggregation by increasing cyclic AMP levels. | Expressed by fibroblasts, follicular dendritic cells, endothelial cells, smooth muscle cells, and thymic nurse cells etc. | (22,23) |
|  | **Pro-fibrinolytic factors** | | | |
| 14 | Tissue-type plasminogen activator (tPA) | Activator of plasminogen into plasmin resulted in fibrin degradation | Mainly expressed by endothelial cells but also by hepatocytes, brain cells, smooth muscle cells, epithelial cells, and immune cells | (24–27) |
| 15 | Urokinase-type plasminogen activator | Fibrinolytic system, activation of plasminogen into plasmin resulted in fibrin degradation | Secreted in an inactive form by many cells, such as those of the urogenital system, leukocytes, fibroblasts, endothelial cells and tumor cells | (28) |
|  | **Anti-fibrinolytic factors** | | | |
| 16 | Plasminogen activator inhibitor-1 (PAI-1) | Inhibitors of plasminogen activators to suppress fibrin degradation | Endothelial cells, megakaryocytes and leukocytes, smooth muscle cells, fibroblasts, adipocytes, hepatocytes, etc. PAI-1 is stored in platelets, secreted to blood flow, or on the subendothelial matrix. | (29) |
| 17 | PAI-2 | Inhibitors of tissue-type and urokinase-type plasminogen activators to suppress fibrin degradation | Not detectable in plasma from men and from nonpregnant women, upregulated by cells in the placenta, monocytes/macrophages,  fibroblasts, and neurons upon stimulation | (29,30) |
| 18 | Carboxypeptidase U (also known as thrombin activatable fibrinolysis inhibitor (TAFI)) | Inhibitor of fibrinolysis upon activation by thrombin or thrombin-thrombomodulin complex | Produced by the liver | (31) |
|  | **Inhibitors of both coagulant and fibrinolytic factors** | | | |
| 19 | PAI-3 | Inhibitor of activated protein C and other factors of the coagulation cascade (thrombin, FXa, FIXa, and thrombin-thrombomodulin) and the fibrinolysis system (urokinase) | Expressed in many tissues in the liver, kidney, spleen, pancreas, and reproductive organs | (32,33) |
| 20 | Alpha-2-Macroglobulin | Inhibitor of thrombin, FXa, activated protein C, plasmin, tissue-plasminogen activator, and urokinase 🡪 balanced regulation of coagulation and fibrinolysis | Mainly expressed in the liver and by macrophages and fibroblasts at lower levels | (34,35) |

**References**

1. Mojiri A, Stoletov K, Lorenzana Carrillo MA, Willetts L, Jain S, Godbout R, et al. Functional assessment of von Willebrand factor expression by cancer cells of non-endothelial origin. Oncotarget. 2017 Feb 21;8(8):13015–29.

2. Peyvandi F, Garagiola I, Baronciani L. Role of von Willebrand factor in the haemostasis. Blood Transfus. 2011;s3–8.

3. Colognato H, Yurchenco PD. Form and function: The laminin family of heterotrimers. Dev Dyn. 2000 May 24;218(2):213–34.

4. Yap L, Tay HG, Nguyen MTX, Tjin MS, Tryggvason K. Laminins in Cellular Differentiation. Trends Cell Biol. 2019 Dec;29(12):987–1000.

5. Sherbet GV. Thrombospondins. In: Growth Factors and Their Receptors in Cell Differentiation, Cancer and Cancer Therapy [Internet]. Elsevier; 2011 [cited 2023 Oct 12]. p. 111–4. Available from: https://linkinghub.elsevier.com/retrieve/pii/B9780123878199000104

6. Andrews RK, Berndt MC. The GPIb-IX-V Complex. In: Platelets [Internet]. Elsevier; 2013 [cited 2023 Oct 12]. p. 195–213. Available from: https://linkinghub.elsevier.com/retrieve/pii/B9780123878373000109

7. Vilar R, Fish RJ, Casini A, Neerman-Arbez M. Fibrin(ogen) in human disease: both friend and foe. Haematologica. 2020 Feb;105(2):284–96.

8. Ruggeri ZM, Jackson SP. Platelet Thrombus Formation in Flowing Blood. In: Platelets [Internet]. Elsevier; 2013 [cited 2023 Oct 12]. p. 399–423. Available from: https://linkinghub.elsevier.com/retrieve/pii/B9780123878373000201

9. Yasumitsu H, Seo N, Misugi E, Morita H, Miyazaki K, Umeda M. Vitronectin secretion by hepatic and non-hepatic human cancer cells. Vitro Cell Dev Biol - Anim. 1993 May;29(5):403–7.

10. Ponczek MB. High Molecular Weight Kininogen: A Review of the Structural Literature. Int J Mol Sci. 2021 Dec 13;22(24):13370.

11. Manon‐Jensen T, Kjeld NG, Karsdal MA. Collagen‐mediated hemostasis. J Thromb Haemost. 2016 Mar;14(3):438–48.

12. Adams SL. Collagen Gene Expression. Am J Respir Cell Mol Biol. 1989 Sep;1(3):161–8.

13. Leblond CP. Synthesis and secretion of collagen by cells of connective tissue, bone, and dentin. Anat Rec. 1989 Jun;224(2):123–38.

14. Protty MB, Jenkins PV, Collins PW, O’Donnell VB. The role of procoagulant phospholipids on the surface of circulating blood cells in thrombosis and haemostasis. Open Biol. 2022 Apr;12(4):210318.

15. Rao LVM, Pendurthi UR. Regulation of tissue factor coagulant activity on cell surfaces: *Tissue factor*. J Thromb Haemost. 2012 Nov;10(11):2242–53.

16. Clark SR, Thomas CP, Hammond VJ, Aldrovandi M, Wilkinson GW, Hart KW, et al. Characterization of platelet aminophospholipid externalization reveals fatty acids as molecular determinants that regulate coagulation. Proc Natl Acad Sci. 2013 Apr 9;110(15):5875–80.

17. Castellino FJ, Ploplis VA. The protein C pathway and pathologic processes. J Thromb Haemost. 2009 Jul;7:140–5.

18. Dahlbäck B, Villoutreix BO. The anticoagulant protein C pathway. FEBS Lett. 2005 Jun 13;579(15):3310–6.

19. Gierula M, Ahnström J. Anticoagulant protein S—New insights on interactions and functions. J Thromb Haemost. 2020 Nov;18(11):2801–11.

20. Lemke G, Rothlin CV. Immunobiology of the TAM receptors. Nat Rev Immunol. 2008 May;8(5):327–36.

21. Broze Jr. G J. Tissue factor pathway inhibitor: structure-function. Front Biosci. 2012;17(1):262.

22. Noe L, Peeters K, Izzi B, Van Geet C, Freson K. Regulators of Platelet cAMP Levels: Clinical and Therapeutic Implications. Curr Med Chem. 2010 Sep 1;17(26):2897–905.

23. Dorris SL, Peebles RS. PGI _2_ as a Regulator of Inflammatory Diseases. Mediators Inflamm. 2012;2012:1–9.

24. Hart P, Burgess D, Vitti G, Hamilton J. Interleukin-4 stimulates human monocytes to produce tissue-type plasminogen activator. Blood. 1989 Sep 1;74(4):1222–5.

25. Louessard M, Lacroix A, Martineau M, Mondielli G, Montagne A, Lesept F, et al. Tissue Plasminogen Activator Expression Is Restricted to Subsets of Excitatory Pyramidal Glutamatergic Neurons. Mol Neurobiol. 2016 Sep;53(7):5000–12.

26. Stevenson TK, Lawrence DA. Characterization of Tissue Plasminogen Activator Expression and Trafficking in the Adult Murine Brain. eneuro. 2018 Jul;5(4):ENEURO.0119-18.2018.

27. Zheng Z, Nayak L, Wang W, Yurdagul A, Wang X, Cai B, et al. An ATF6-tPA pathway in hepatocytes contributes to systemic fibrinolysis and is repressed by DACH1. Blood. 2019 Feb 14;133(7):743–53.

28. The urokinase-system – role of cell proliferation and apoptosis. Histol Histopathol. 2007 Nov 14;(23):227–36.

29. Zorio E, Gilabert-Estelles J, Espana F, Ramon L, Cosin R, Estelles A. Fibrinolysis: The Key to New Pathogenetic Mechanisms. Curr Med Chem. 2008 Apr 1;15(9):923–9.

30. Medcalf RL. Plasminogen Activator Inhibitor Type 2. In: Methods in Enzymology [Internet]. Elsevier; 2011 [cited 2023 Oct 12]. p. 105–34. Available from: https://linkinghub.elsevier.com/retrieve/pii/B9780123864710000067

31. Willemse JL, Heylen E, Nesheim ME, Hendriks DF. Carboxypeptidase U (TAFIa): a new drug target for fibrinolytic therapy? J Thromb Haemost. 2009 Dec;7(12):1962–71.

32. Meijers JCM, Marquart JA, Bertina RM, Bouma BN, Rosendaal FR. Protein C inhibitor (plasminogen activator inhibitor-3) and the risk of venous thrombosis: Protein C Inhibitor and Venous Thrombosis. Br J Haematol. 2002 Aug;118(2):604–9.

33. Suzuki K. Protein C inhibitor (PAI-3): structure and multi-function. Fibrinolysis Proteolysis. 2000 Mar;14(2–3):133–45.

34. Lagrange J, Lecompte T, Knopp T, Lacolley P, Regnault V. Alpha‐2‐macroglobulin in hemostasis and thrombosis: An underestimated old double‐edged sword. J Thromb Haemost. 2022 Apr;20(4):806–15.

35. Vandooren J, Itoh Y. Alpha-2-Macroglobulin in Inflammation, Immunity and Infections. Front Immunol. 2021 Dec 14;12:803244.
