# Supplementary figures and images for: Impact of tissue factor expression and administration routes on thrombosis development induced by mesenchymal stem/stromal cell infusions: re-evaluating the dogma
Source: Stem Cell Res Ther. 2024 Feb 27;15:56. doi: 10.1186/s13287-023-03582-3 (PMC10900728; doi:10.1186/s13287-023-03582-3)

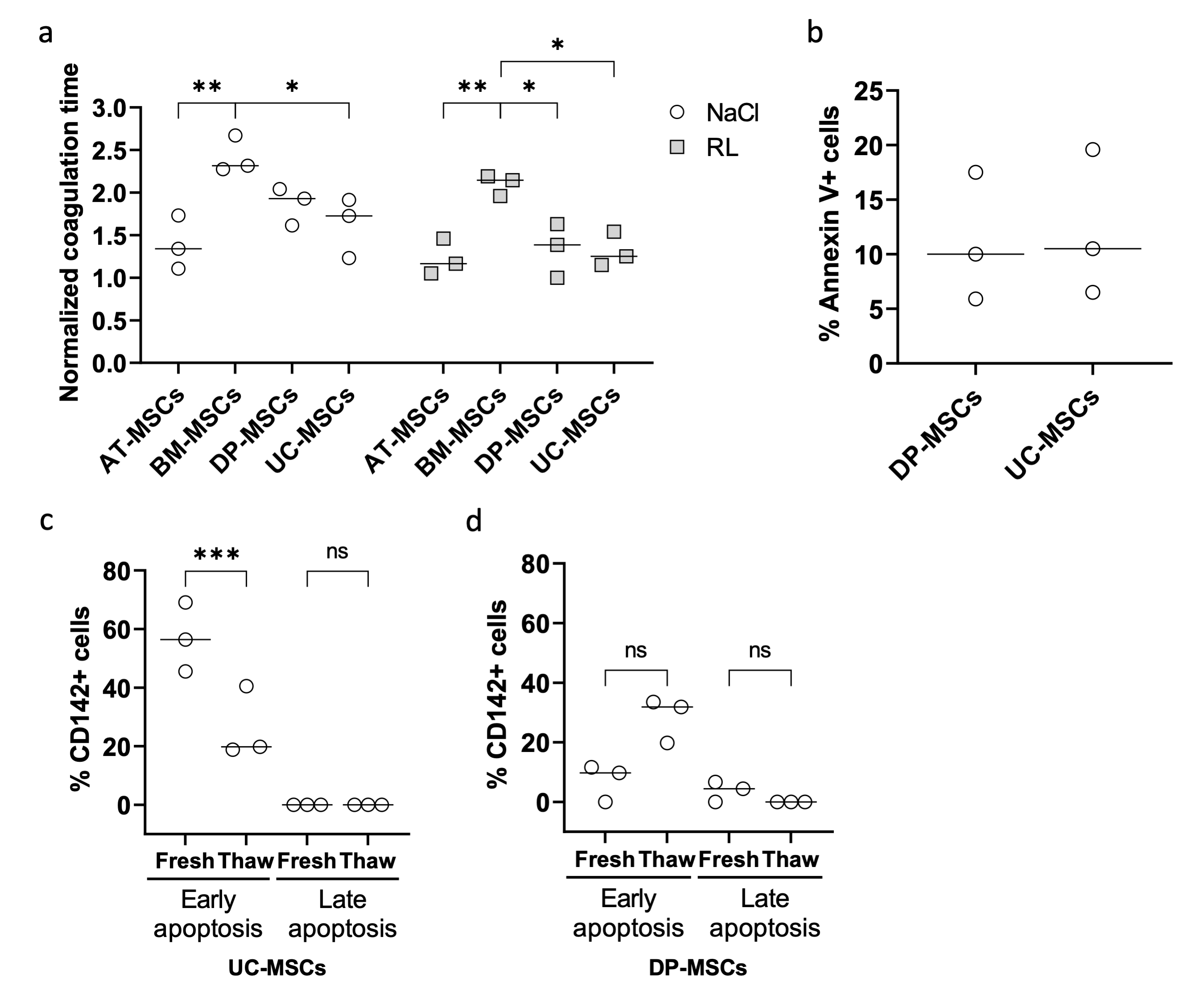

Supplement: Supplementary file 2 — Additional file 2: Figure S1. Analysis of the in vitro MSC-induced clotting and levels of phosphatidylserine (PS) and TF on MSCs. a Comparative study of the coagulant activity of MSCs derived from AT, BM, DP and UC. b Detection of Annexin V binding PS on the surface of DP- and UC-MSCs by flow cytometry. c and d Expression of TF in the Annexin V+7-AAD-early apoptotic and Annexin V+7-AAD+ late apoptotic cell populations. [file 13287_2023_3582_MOESM2_ESM.tiff]

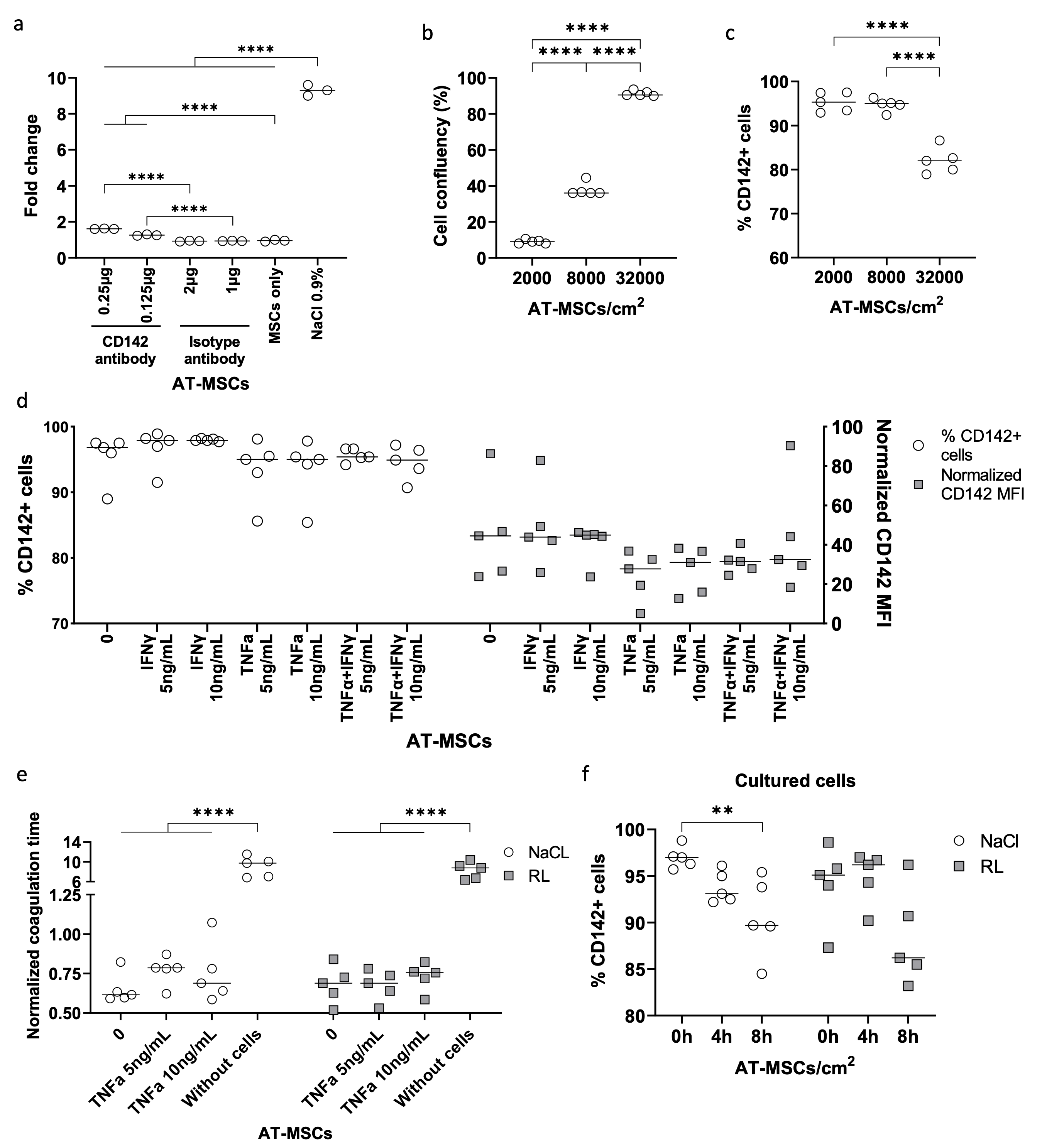

Supplement: Supplementary file 3 — Additional file 3: Figure S2. TF expression and activity on AT-MSCs. a Treatment of AT-MSCs with the anti-TF inhibitory antibody (clone HTF-1) resulted in a partial reduction in their coagulant activity. b-f Impact of culture conditions on TF expression and activity in AT-MSCs. b AT-MSCs were seeded at three different concentrations, and their cell density was measured. c The corresponding TF expression levels were analyzed, showing lower TF expression at the highest cell density. d AT-MSCs did not change TF expression in the presence of the inflammatory cytokines INFg and TNFa. e Furthermore, both AT-MSCs and their TNFa-treated counterparts indicated comparable coagulation activity when incubated with healthy plasma. f Storage of AT-MSCs might reduce TF levels. [file 13287_2023_3582_MOESM3_ESM.tiff]

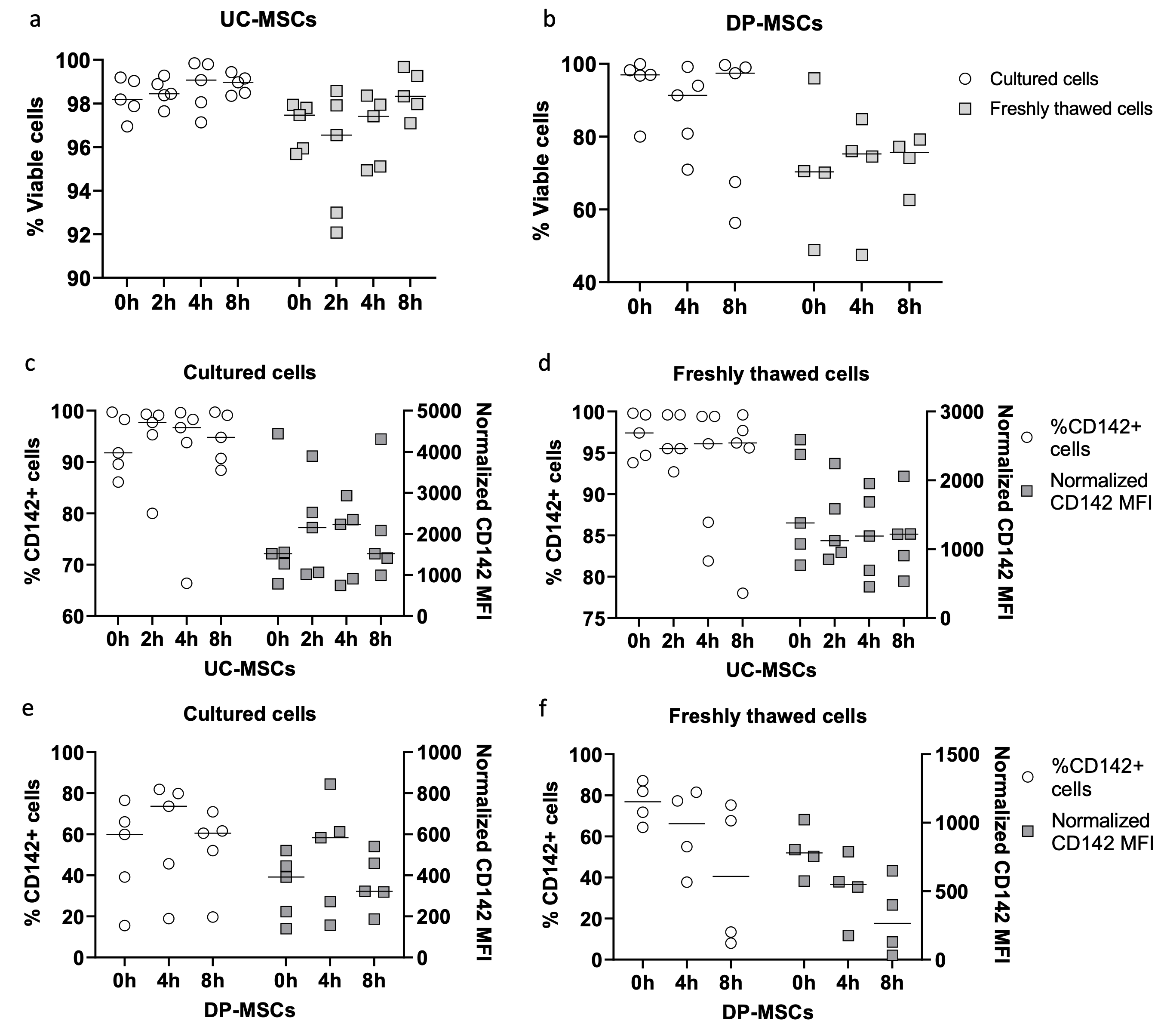

Supplement: Supplementary file 4 — Additional file 4: Figure S3. Impact of storage on the survival and TF expression of UC- and DP-MSCs. a and b The survival rate of UC-MSCs (a) and DP-MSCs (b) was observed for up to 8 h storage in NaCl. c–f TF expression of cultured UC-MSCs (c), freshly thawed UC-MSCs (d), cultured DP-MSCs (e), and freshly thawed UC-MSCs (f) within 8 h of storage in RL is depicted. [file 13287_2023_3582_MOESM4_ESM.tiff]
